# GRADE-ADOLOPMENT of hyperthyroidism treatment guidelines for a Pakistani context

**DOI:** 10.1186/s12902-023-01493-1

**Published:** 2024-03-21

**Authors:** Russell Seth Martins, Sarah Nadeem, Abeer Aziz, Sajjan Raja, Alina Pervez, Najmul Islam, Asma Ahmed, Aisha Sheikh, Saira Furqan, Nanik Ram, Azra Rizwan, Nashia Ali Rizvi, Mohsin Ali Mustafa, Salima Saleem Aamdani, Bushra Ayub, Muhammad Qamar Masood

**Affiliations:** 1 Center for Clinical Best Practices, Clinical and Translational Research Incubator (CITRIC), Aga Khan University, Karachi, 74800 Pakistan; 2 Section of Endocrinology, Department of Medicine, Aga Khan University, Karachi, 74800 Pakistan; 3 FACE (Fellow American College of Endocrinology), Internal Medicine & Endocrinology, Diabetes & Metabolism, Internal Medicine, and Endocrinology, Women in Medicine Committee, Associate Dean’s Women Faculty Forum, Aga Khan University, Karachi, Pakistan; 4 Medical College, Aga Khan University, Karachi, 74800 Pakistan; 5 Department of Medicine, Aga Khan University, Karachi, 74800 Pakistan; 6 Learning Research Centre, Patel Hospital, Karachi, 75300 Pakistan

**Keywords:** Guidelines, LMIC, Pakistan, GRADE, Adolopment, Thyroid

## Abstract

**Introduction:**

The prevalence of hyperthyroidism in Pakistan is 2.9%, which is two times higher than in the United States. Most high-quality hyperthyroidism clinical practice guidelines (CPGs) used internationally originate from high-income countries in the West. Local CPGs in Pakistan are not backed by transparent methodologies. We aimed to produce comprehensive, high-quality CPGs for the management of hyperthyroidism in Pakistan.

**Methods:**

We employed the GRADE-ADOLOPMENT approach utilizing the *2016 American Thyroid Association Guidelines for Diagnosis and Management of Hyperthyroidism and Other Causes of Thyrotoxicosis* as the source CPG. Recommendations from the source guideline were either adopted as is, excluded, or adapted according to our local context.

**Results:**

The source guideline included a total of 124 recommendations, out of which 71 were adopted and 49 were excluded. 4 recommendations were carried forward for adaptation via the ETD process, with modifications being made to 2 of these. The first addressed the need for liver function tests (LFTs) amongst patients experiencing symptoms of hepatotoxicity while being treated with anti-thyroid drugs (ATDs). The second pertained to thyroid status testing post-treatment by radioactive iodine (RAI) therapy for Graves’ Disease (GD). Both adaptations centered around the judicious use of laboratory investigations to reduce costs of hyperthyroidism management.

**Conclusion:**

Our newly developed hyperthyroidism CPGs for Pakistan contain two context-specific modifications that prioritize patients’ finances during the course of hyperthyroidism management and to limit the overuse of laboratory testing in a resource-constrained setting. Future research must investigate the cost-effectiveness and risk-benefit ratio of these modified recommendations.

**Supplementary Information:**

The online version contains supplementary material available at 10.1186/s12902-023-01493-1.

## Introduction

Hyperthyroidism is a common endocrine condition that presents a significant global challenge with high morbidity and mortality rates [1, 2]. In Pakistan, a South Asian lower-middle-income country (LMIC) with a population of over 220 million [3], the prevalence is 2.9% [4]. This is more than two times higher than the United States of America (US: 1.3% [5]) and more than three times higher than in Europe (0.8% [6]). The high prevalence of hyperthyroidism in Pakistan can be attributed to a complex interplay of factors, with key determinants including geographical variables and ethnic diversity [7]. Hyperthyroidism confers an increased all-cause mortality risk, particularly due to cardiovascular causes [8].

Evidence-based clinical practice guidelines (EBCPGs) direct the diagnosis and management of hyperthyroidism, so as to achieve standardization of favorable clinical outcomes [9, 10]. EBCPGs created by institutions in developed countries in the West, such as the US [11] and European countries [12], are oftentimes adopted by other countries, particularly LMICs, for use in their settings. This is because LMICs, like Pakistan, usually lack the research infrastructure and financial resources to independently develop EBCPGs de novo for their own healthcare context [13]. However, the application of such EBCPGs for the management of hyperthyroidism in Pakistan presents a problem, as the country’s landscape differs due to several factors [14]. These include disease epidemiology [15], healthcare financing [16], dietary habits and iodine consumption [17], socio-economic influences [18], and disease-related awareness [19]. Therefore, it becomes imperative for an LMIC like Pakistan to create EBCPGs that best suit the unique context of the setting where they will be applied.

In cases where the de novo creation of EBCPGs is not practically feasible, a process called “adolopment” provides a suitable alternative. Adolopment describes a combination of adoption (verbatim use), adaptation (contextual modifications), and de novo development, thus leveraging the benefits of pre-existing high-quality EBCPGs while ensuring local appropriateness. The GRADE-ADOLOPMENT method [13], developed by GRADE (Grading of Recommendations Assessment, Development, and Evaluation), is a globally accepted and implemented process of EBCPG adolopment. It uses evidence-to-decision (ETD) tables, which summarize the best available evidence on a topic, to guide decisions regarding the need for contextual modifications of individual recommendations within an EBCPG [20]. GRADE-ADOLOPMENT has been used in countries and regions across the world, including Saudi Arabia [13], Australia [21], Tunisia [22], the Eastern Mediterranean region [23], the Asia-Pacific region [24], Mexico [25], and the United Kingdom [26].

Although the Pakistan Endocrine Society, founded in 2003, is involved in the creation of local EBCPGs for the management of common endocrine disorders in Pakistan, their publications have thus far focused on diabetes mellitus and metabolic syndrome [27]. Moreover, the processes involved in the development of these EBCGPs are not explicitly described. Consequently, there is immense need for local hyperthyroidism EBCPGs to be developed following a transparent, standardized process that makes use of existing available best-evidence EBCPGs with appropriate context-specific modifications. Such EBCPGs would bring the healthcare system of Pakistan a step closer to achieving optimal health outcomes in hyperthyroidism and would gain credibility by virtue of their transparent development processes. Thus, we aimed to employ the GRADE-ADOLOPMENT process to develop local evidence-based EBCPGs for the management of hyperthyroidism in adults by GPs in Pakistan.

## Methodology

### Setting

This process was conducted at the CITRIC (Clinical and Translational Research Incubator) Center for Clinical Best Practices (CCBP) at the Aga Khan University (AKU), Pakistan. The AKU is a private sector, not-for‐profit hospital in Pakistan, and is also the country’s leading healthcare and biomedical research facility [28].

The CITRIC CCBP at AKU is tasked with the adaptation and development of EBCPG and care pathways to standardize and improve healthcare in Pakistan. The GRADE-ADOLOPMENT processes described in this study have been implemented by the CCBP, in collaboration with the expertise of the Section of Endocrinology at AKU and the GRADE-USA working group, in the development of hyperthyroidism management EBCPGs for use by general practitioners (GPs)/primary care physicians in Pakistan. The decision to create hyperthyroidism EBCPGs for GPs rather than specialist endocrinologists is due to the lack of access to specialists in Pakistan [29].

### Study team

The study team is comprised of the CCBP research staff (who are proficient in GRADE methodology and in the development of EBCPGs) as well as endocrinology faculty led by Endocrinology Section Head of AKU.

### Source guideline selection

The source guideline is the single, original, “parent” EBCPG that undergoes the GRADE-ADOLOPMENT process in the development of a local EBCPG. The *2016 American Thyroid Association Guidelines for Diagnosis and Management of Hyperthyroidism and Other Causes of Thyrotoxicosis *[30] was selected by the Section of Endocrinology as the source EBCPG, due to its comprehensive set of recommendations, integrated approach to management, and high-quality synthesis of available evidence. The 2016 American Thyroid Association source guideline used the GRADE approach for the strength of the recommendations and the quality of evidence.

### Guideline review

**Fig. 1:**
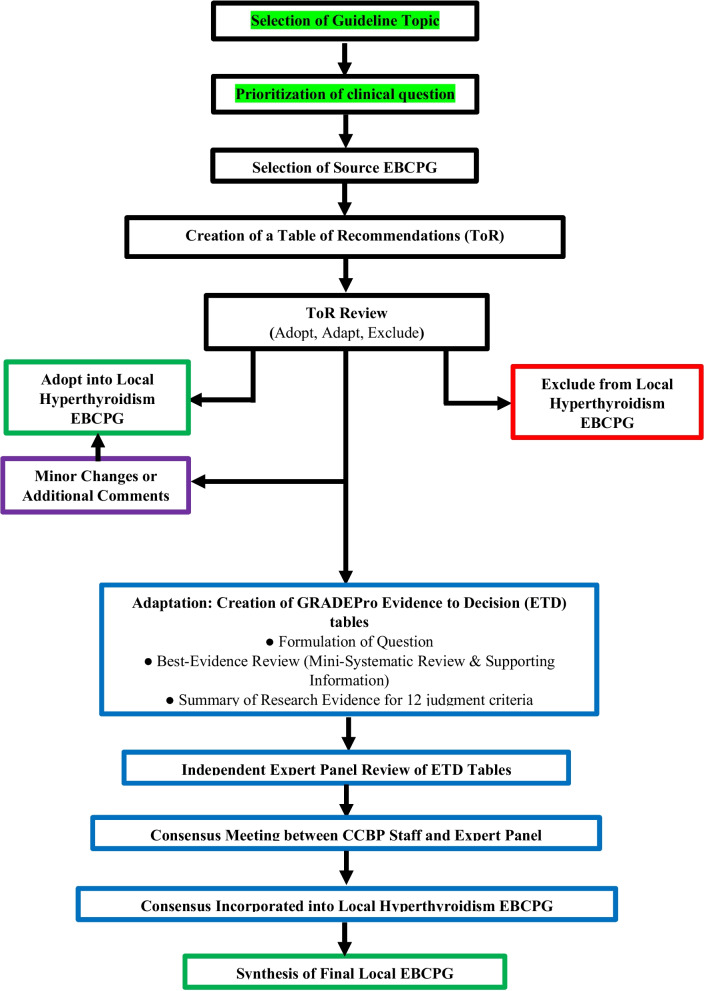
GRADE-ADOLOPMENT process for Hyperthyroidism Management EBCPG for Pakistan

Figure 1 delineates the adolopment process used in our study. First, a Table of Recommendations (ToR) was created by extracting and compiling all recommendations mentioned in the source EBCPG. Two senior attending endocrinologists reviewed the ToR independently and marked each recommendation as either “*Adopt*,” “*Adapt”* or “*Exclude*.” Discrepancies were settled in consensus with the Section Head of Endocrinology. Recommendations marked “*Adopt*” were incorporated as is or with minor changes into the local EBCPG, while those marked “*Exclude*” were omitted from the local EBCPG. Exclusion was based on the recommendation pertaining to pediatric or inpatient management, or if the recommendation was deemed irrelevant to the local Pakistani context. Other reasons for exclusion were required to be explained by the reviewers.

Recommendations marked “*Adapt*” were deemed to warrant additional review and revision via the GRADE-ADOLOPMENT process (detailed below) before incorporation into the local EBCPGs. Our adolopment process (Fig. 1) had two important differentiations to the one described originally [13]. Firstly, we did not create any recommendations de novo, which was due to a lack of perceived need for additional recommendations. Secondly, recommendations that were deemed to require only minor and straightforward changes prior to adoption were not subjected to the complete adaptation process consisting of ETD tables and expert panel review.

### GRADEPro evidence to decision Framework

GRADEPro is a web application used to help create, manage, and share summaries of research evidence [31]. The CCBP staff involved in this study underwent a training module to master use of GRADEPro for the GRADE-ADOLOPMENT process. The software was used to develop Evidence to Decision (ETD) tables to reach a consensus on each of the recommendations marked “*Adapt*.”

ETD tables that summarize evidence to enable members of an expert panel to make healthcare recommendations or decisions. Development of ETD tables begins with formulation of a question structured as follows: “Should the *Intervention/Suggested Change* be favored over the *Comparison/Current Standard of Practice?*” The pros and cons of the suggested change are judged by an expert panel across 12 criteria, that are shown in Supplementary Table 1 (Additional file 1).

Each criterion was supported with evidence gathered through a best evidence review process (Additional file 1), to provide local context for the pros and cons of the recommendation. The CCBP team summarized the newly gathered evidence for each criterion in the “*Research Evidence*” and “*Additional Considerations*” columns. The GRADE-USA working group was deeply involved in the creation of the ETD tables.

### Expert panel review

An expert panel of five endocrinology faculty from AKU were invited by the Endocrinology Section Head to review the completed ETD table for each recommendation and provide their judgement for each criterion. This judgment was in the form of a single selection from multiple response options. If, for any criteria, an expert required additional evidence, they informed the CCBP team. An effort was made to source the requisite information, which, if found, was shared with all the panel members. Experts’ judgements were sought in an anonymous and confidential manner, with the GRADEPro software allowing reviewers to select options and provide feedback without their identity known to fellow experts or the CCBP team. A sample of a GRADEPro ETD is shown as Supplementary Table 2 (Additional file 1).

### Final recommendation revisions & synthesis

Once all the members of the expert panel had provided their responses to the ETD, the CCBP staff synthesized their responses to produce a summary of judgments. The CCBP staff conducted a meeting with the expert panel to review the summary of judgments and reach a final unanimous consensus on the need for and nature of any revisions to the recommendations in question. The strength of each recommendation was also decided. Finally, the consensus was presented to the Section Head of Endocrinology for review, after which the recommendation was incorporated into the Pakistani EBCPG with a summary of the consensus decision.

### Final debriefing to identify challenges & explore solutions

Two focus group discussions (FGDs) were conducted to identify challenges faced throughout the entire GRADE-ADOLOPMENT process and to explore corresponding solutions. These FGDs were led by a member of the CCBP team and included the CCBP staff and the Section Head of Endocrinology. Participants were given the opportunity to first brainstorm challenges and solutions independently, and these were then discussed within the FGD. Each challenge was decided as per consensus opinion to be either a major or minor challenge. The CCBP team then categorized the final list of specific challenges within broad themes, and their corresponding solutions were presented alongside them.

## Results

### Initial review of source guideline

**Fig. 2:**
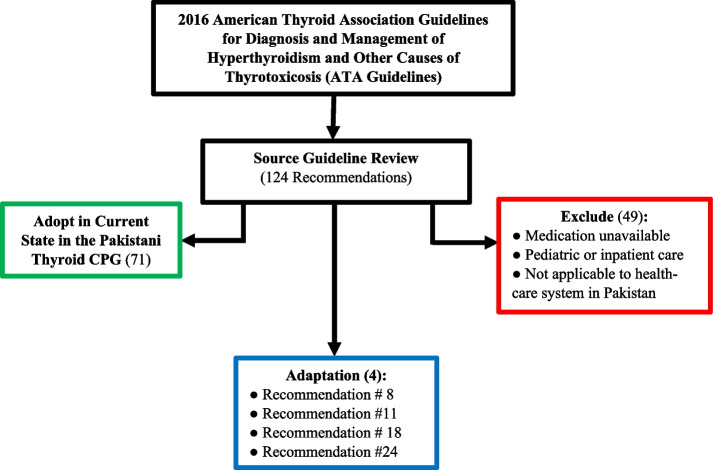
Outcomes of table of recommendations review

The source guideline included a total of 124 recommendations, out of which 71 were adopted and 49 were excluded. 4 recommendations were carried forward for adaptation via the ETD process (Fig. 2) (Supplementary Table 3) (Additional file 1). A list of all excluded recommendations can be found in Supplementary Table 4 (Additional file 1).

### Evidence-to-decision (ETD) tables

Amongst the four recommendations that underwent the adaptation process, modifications were made to two (Tables 1 and 2), while the remaining two were unchanged (Tables 3 and 4). The complete Evidence to Decision tables with the summary of judgements for the modified recommendations can be found in Supplementary Tables 5 & 6 (Additional files 2 & 3).

### Challenges and solutions

The challenges faced were broadly categorized into four main themes: resources, stakeholder support and involvement, resistance to change, and methodological limitations (Table 5). Solutions proposed for the challenges faced will be incorporated in the future updates of the guideline.

**Table 1:** Summary of ETD for Recommendation # 8

**Original Recommendation**	Sufficient activity of RAI should be administered in a single application, typically a mean dose of 10–15 mCi (370–555 MBq), to render the patient with GD hypothyroid (Strong Recommendation; Moderate Quality Evidence)			
**Modified Recommendation**	As a substitute for RAI, ATD treatment (with routine thyroid function test monitoring) may be continued or surgery may be performed, to render the patient with GD hypothyroid			
**Overall Conclusion**				
☐Strong recommendation for Modification	☐Conditional recommendation for Proposed Modification	☐Conditional recommendation for either Original Recommendation or Proposed Modification	☐Conditional recommendation for Original Recommendation	☒Strong recommendation for Original Recommendation
**Additional Suggestions**: • If no major financial concerns, RAI therapy is a feasible option as a definitive treatment in patients on high doses of ATDs for GD. • RAI therapy should be preferred over surgical treatment in GD patients not responding to medical treatment. • Patients with uncontrolled GD without orbitopathy, relapsed cases, and those requiring ATD for more than 2 years should also be considered for RAI therapy				
**Justification**: • Long-term follow up is reduced as once the patient becomes hypothyroid thyroxine dosage usually remains static. • Expertise for ATD calculation is usually lacking in LMICs, with dose calculation increasing patient-borne costs • ATDs result in a longer time taken to achieve euthyroid status and treatment failure rates are higher compared to RAI therapy. • It is easier to manage primary hypothyroidism as opposed to GD, particularly in the case of non-compliance or potentially deadly thyroid storm. • RAI therapy is more cost-effective than surgical treatment and usually leads to definitive cure.				

**Table 2:** Summary of ETD for Recommendation # 11

**Original Recommendation**	Follow-up within the first 1–2 months after RAI therapy for GD should include an assessment of free T4, total T3, and TSH. Biochemical monitoring should be continued at 4–6-week intervals for 6 months, or until the patient becomes hypothyroid and is stable on thyroid hormone replacement (Strong Recommendation; Low-Quality Evidence)			
**Modified Recommendation**	Follow-up within 1–2 months after RAI therapy should include an assessment of free T4 only. Once FT4 is found to be low then a TSH should be checked. When TSH is high, that means post-radioiodine hypothyroidism has been achieved, and levothyroxine replacement for the patient should be started at approximate dosage 1.6 µg/kg, then recheck TSH no earlier than 6-8-weeks to assess adequacy of dose. FT4 should be monitored every 4–6 weeks for a duration of 6 months after RAI until it falls within the hypothyroid range. Once hypothyroid & on stable dose of levothyroxine, TSH should be measured every 6–12 months. (Conditional Recommendation; Low-Quality Evidence)			
**Overall Conclusion**				
☐Strong recommendation for Modified Recommendation	☒Conditional recommendation for Modified Recommendation	☐Conditional recommendation for either Original Recommendation or Modified Recommendation	☐Conditional recommendation for Original Recommendation	☐Strong recommendation for Original Recommendation
**Additional Suggestions**:• The frequency of TFTs can be decreased with time, to be decided on a case-by-case basis.• Timely monitoring of FT4 and initiation of thyroid hormone replacement as soon as the patient becomes hypothyroid will be beneficial to the patient. Once patient is on thyroid hormone replacement, TSH can be checked periodically for assessing adequacy of thyroid hormone replacement.• Public and private lab and hospitals/centers should consider combining RAI administration for thyrotoxicosis with its follow-up laboratory testing in a single package to promote compliance to follow-up testing.				
**Justification**:• The results of TFTs at 1 month may be confusing for the clinician and may result in unnecessary treatment with thioamides.• FT4 is sufficiently sensitive for the purpose of follow-up testing. There is unlikely to be a major difference in outcomes with use of the FT4 alone instead of the full panel.				

**Table 3:** Summary of ETD for Recommendation # 18

**Original Recommendation**	Liver function and hepatocellular integrity should be assessed in patients taking MMI or PTU who experience pruritic rash, jaundice, light-colored stool or dark urine, joint pain, abdominal pain or bloating, anorexia, nausea, or fatigue (Strong Recommendation; Low Quality Evidence).			
**Modified Recommendation**	Liver function tests and hepatocellular integrity should be assessed in patients taking MMI or PTU who experience specific symptoms (pruritic rash, jaundice, light-colored stool or dark urine, abdominal pain) or multiple non-specific symptoms (joint pain, bloating, anorexia, nausea, or fatigue) using only ALT instead of a full LFT panel (alanine transaminase, aspartate transaminase, alkaline phosphatase, gamma-glutamyl transferase; total, conjugated and unconjugated bilirubin) routinely (Strong Recommendation; Low Quality Evidence).			
**Overall Conclusion**				
☒Strong recommendation for Modified Recommendation	☐Conditional recommendation for Modified Recommendation	☐Conditional recommendation for either Original Recommendation or Modified Recommendation	☐Conditional recommendation for Original Recommendation	☐Strong recommendation for Original Recommendation
**Additional Suggestions**: • Remain wary that checking ALT only may lead to missed diagnosis of cholestasis. • In patients with prior history of liver disease or high suspicion of liver damage (multiple specific symptoms), then baseline full LFTs should be performed. • Following drug withdrawal, LFTs need to be repeated after 1–2 weeks				
**Justification**: • The magnitude of liver dysfunction with ATDs in clinical practice is limited, usually occurring with intercurrent illness.				

**Table 4:** Summary of ETD for Recommendation # 24

**Original Recommendation**	If surgery is chosen as treatment for GD, patients should be rendered euthyroid prior to the procedure with ATD pretreatment, with or without beta-adrenergic blockade. A potassium iodide containing preparation should be given in the immediate preoperative period (Strong Recommendation; Low Quality Evidence).			
**Modified Recommendation**	If surgery is chosen to manage thyrotoxicosis, patients should be rendered euthyroid prior to the procedure with ATD pre-treatment and/or beta-adrenergic blockade. A KI-containing preparation should be given in the immediate preoperative period.			
**Overall Conclusion**				
☐Strong recommendation for Modified Recommendation	☐Conditional recommendation for Modified Recommendation	☐Conditional recommendation for either Original Recommendation or Modified Recommendation	☐Conditional recommendation for Original Recommendation	☒Strong recommendation for Original Recommendation
**Additional Suggestions**: • Potassium iodide should be administered one hour after ATD to prevent iodine being used as substrate for further thyroid hormone synthesis				
**Justification**: • Rendering a patient euthyroid prior to surgery is essential. Potassium iodide helps achieve euthyroid status while also providing additional potential benefits such as decreasing thyroid vascularity and limiting intraoperative bleeding.				

**Table 5:** Challenges faced and proposed solutions

Category of Challenge	Specific Challenge	Proposed Solution
**Resources**	• Suboptimal original data from Pakistan^a^	• Make use of regional literature • Judicious use of grey-literature
	• Structuring the GRADE-ADOLOPMENT process to the resource-constrained context of Pakistan (revise through experience, highlight resource gap)^b^	**•** Conduct a thorough, realistic resource assessment, and highlight resource gaps • Revise process accordingly through experience
	• Inadequate manpower/size of workforce^a^	**•** Involve students and trainees on a volunteer basis
**Stakeholder Support & Involvement**	• Suboptimal departmental support^b^	• Involve all stakeholders from the start • Emphasize and reiterate mutual interests • Design specific curricula for all stakeholders involved • Tailor and deliver presentations to all stakeholders involved • Collaborate with patient advocacy groups • Community outreach and education • Involve Medical Societies for review of guidelines • Invite external topic specialists
	• Suboptimal provincial/federal government involvement^a^	
	• Suboptimal involvement of external societies or organizations^a^	
	• Absence of patients’ perspective^a^	
	• Absence of general practitioners’ perspective^a^	
	• Absence of the allied health perspective^a^	
**Resistance to Change**	• Experts’ doubts regarding need for local EBCPGs^b^	• Initial presentation to emphasize need for local EBCPGs, robustness of the GRADE-ADOLOPMENT process, and the importance of strict adherence to rigorous GRADE-ADOLOPMENT processes in order to produce credible guidelines
	**•** Experts’ exercising caution/opting for middle-ground with regards to decision-making in ETD table^b^	• Emphasize the anonymity of the ETD process • Emphasize importance of incorporating varying schools of thought in the adaptation process
	• Experts’ doubts regarding nationwide implementation of local guidelines^a^	• Involve decision-makers from other institutions across the country and ensure buy-in to the newly adoloped EBCPGs
**Methodological Limitations**	• Individual-level biases from experts^b^	• Increase the number and diversity of experts • Gauge acceptability and accuracy of any revisions made by getting feedback from experts from external institutes
	• Group-level biases from experts^b^	
	• Suboptimal generalizability of consensus opinion based on 5 experts^b^	
	• Expert opinion is no substitute for the lack of scientific evidence^a^	• Supplement the expert opinion with as much auxiliary evidence as possible • Plan future studies to answer specific questions that arise during the GRADE-ADOLOPMENT process

^a^Minor challenge; ^b^Major Challenge

## Discussion

In this paper, we applied the GRADE-ADOLOPMENT process to the *2016 American Thyroid Association Guidelines for Diagnosis and Management of Hyperthyroidism and Other Causes of Thyrotoxicosis *[30] to adolop EBCPGs for the management of hyperthyroidism in the local context of Pakistan. Out of a total of 124 recommendations, 71 were adopted, 49 were excluded, and 4 were subjected to the process of adaptation. The adapted recommendations primarily focused on accommodating patient-centered factors and accounting for a lack of resources in Pakistan, without a significant compromise in clinical outcomes.

The first adapted recommendation addressed the need for liver function tests (LFTs) amongst patients experiencing symptoms of hepatotoxicity while being treated with anti-thyroid drugs (ATDs). The source EBCPG recommended a full panel of LFTs (alanine transaminase, aspartate transaminase, alkaline phosphatase, gamma-glutamyl transferase; total, conjugated and unconjugated bilirubin) for all patients experiencing any symptoms remotely suggestive of hepatotoxicity (pruritic rash, jaundice, light-colored stool or dark urine, joint pain, abdominal pain or bloating, anorexia, nausea, or fatigue) [30]. However, this recommendation was adapted to advise the use of only alanine transaminase (ALT) to diagnose the extent of liver injury in patients experiencing highly specific symptoms (jaundice, pruritis, or change in stool color). The rationale behind this adaptation was centered around the infrequent incidence of hepatotoxicity while on ATD (1.4–6.3% [32]) and the patient-borne financial ramifications of over-testing. In contrast to high-income countries where government or private insurance covers the majority of healthcare costs, almost 60% of healthcare costs in Pakistan are via out-of-pocket payment by patients [33], with national health coverage provided to only 20% of the population [34]. The cost of a full LFT panel in Pakistan ranges from $3.73–7.15, which is between 3 and 7 times more than a single ALT test (ranges from $1.01–1.67). However, while patient finances must be given full consideration in the management of hyperthyroidism, future research is needed to investigate the cost-effectiveness of the adapted recommendation in a Pakistani population.

The second recommendation to undergo the adaptation process was related to thyroid status testing post-treatment by radioactive iodine (RAI) therapy for Graves’ Disease (GD). The source EBCPG recommends assessing free T4 (FT4), total T3, and thyroid-stimulating hormone (TSH) amongst patients within 1–2 months after patients with GD receive RAI therapy, followed by 4–6 weekly testing for 6 months, or until the patient becomes hypothyroid and is stable on thyroid hormone replacement This recommendation was modified to advise the assessment of only FT4 at initial follow-up, with subsequent TSH assessment only in the case of low T4. The keystone of this modification was the consensus that FT4 alone is a sufficiently sensitive modality to detect post-RAI hypothyroidism, and that TSH suppression in the post-RAI period may limit its accuracy in reflecting thyroid status. In fact, this misleading suppression of TSH after RAI therapy may prompt the physician to initiate thioamides unnecessarily. Moreover, in Pakistan, the use of a single FT4 test (ranges from $4.92–8.30) is about a third the price of a full panel consisting of FT4, T3 and TSH ($12.7–18.1). In fact, a sizeable percentage (48.8%) of the overall management costs for hyperthyroidism are attributable to laboratory testing [35]. Lastly, if both the initial FT4 and subsequent TSH assessment reflect hypothyroidism, and thyroid hormone replacement is initiated and optimized, long-term assessment of treatment effectiveness can be monitored by TSH alone. To facilitate adherence to follow-up and routine post-operative testing, it is recommended that public and private laboratories in Pakistan should partner with healthcare centers to create comprehensive and appropriate care packages which include all post-treatment management and surveillance.

The third recommendation that underwent the adaptation process concerned the preoperative administration of potassium iodide (KI; Lugol’s solution), in addition to ATD and/or beta-blockers, prior to surgical management of GD. While no changes were enacted to this recommendation, experts noted that KI was not widely accessible in Pakistan, with availability of KI being restricted to tertiary care hospitals and large-scale pharmacies, even in urban settings. Though the supporting evidence lacks robustness and clarity, KI is believed to limit intraoperative blood loss by decreasing thyroid vascularity, and also suppress the synthesis and release of thyroid hormone [11]. However, despite these benefits, the lack of widespread availability of KI in Pakistan would undoubtedly preclude its universal use before surgery for GD. Moreover, recent studies have once again called into question the benefits of preoperative KI administration, with regards to its impacts on intraoperative bleeding, difficulty of operation, operative time, and postoperative outcomes [36–38]. Thus, the expert team added an additional comment after adopted recommendation, which reassured readers that a lack of administration of KI would likely not compromise the health outcomes of a patient.

The final recommendation that underwent the adaptation process advised a single dose of RAI to render a patient with GD hypothyroid. Although no modifications were made to the recommendation, discussions centered around the cost-effectiveness and availability of RAI versus an alternate option of employing ATD therapy with regular thyroid function test (TFT) monitoring. However, though ATD therapy may provide a more financially feasible mode of treatment, the remission rate of GD with RAI therapy is significantly higher than with ATD therapy [35]. Therefore, RAI should be considered for definitive treatment in GD patients on high doses of ATD treatment, those not responding to the ATD treatment, and those requiring ATD treatment for more than 2 years.

There are limitations to the GRADE-ADOLOPMENT process used in our study that we would like to acknowledge. Firstly, individual-level (e.g., the Section Head reviewing each ToR to independently to decide whether to adopt, adapt or exclude recommendations) and group-level (e.g., the consensus meeting featuring five experts from a single institution) biases may limit the applicability of our EBCPGs to other settings in Pakistan. Additionally, fundamental to the GRADE-ADOLOPMENT process, the adaptation process was guided primarily by expert consensus, due to the suboptimal availability of local, high-quality level of evidence. Moreover, we limited the inclusion of other important stakeholders, such as patients, allied health professionals, general practitioners, nurses, experts external to AKU, other healthcare centers, external endocrinology organizations or societies, and provincial and federal governments. This was to minimize inevitable delays that would have accompanied a larger team, including logistic difficulties, conflicts of interest, lack of mutual availability, political influences, and lack of direct incentives. However, prior experience in developing such EBCPGs enabled the CCBP team to remain mindful of the needs and values of these groups to a large extent. Lastly, while the efforts to create a local EBCPG for the management of hyperthyroidism have yielded success, the feasibility of widespread utilization and implementation of the EBCPG across Pakistan remains a concern. All the aforementioned limitations represent real-world barriers to the idealistic implementation of the GRADE-ADOLOPMENT process in resource-constrained and poorly structured healthcare systems in LMICs like Pakistan.

## Conclusion

The outcome of the GRADE-ADOLOPMENT process applied to the *2016 American Thyroid Association Guidelines for Diagnosis and Management of Hyperthyroidism and Other Causes of Thyrotoxicosis *[30] yielded two major changes in the newly developed Pakistani EBCPG for the management of hyperthyroidism. These included the recommendation to assess only ALT (as opposed to a full LFT panel) amongst patients on ATDS experiencing symptoms highly specific of hepatotoxicity (as opposed to a higher index of suspicion considering non-specific symptoms like bloating, anorexia, nausea, or fatigue), and the recommendation to assess only FT4 (as opposed to the full panel of FT4, total T3, and TSH) at initial follow-up after RAI therapy for GD, with subsequent TSH assessment only in the case of low T4. The rationale behind both these changes were to prioritize patients’ finances during the course of hyperthyroidism management and to limit the overuse of laboratory testing in a resource-constrained setting. Future research must investigate the cost-effectiveness and risk-benefit ratio of these modified recommendations.

### Supplementary Information

**Additional file 1.**

**Additional file 2.**

**Additional file 3.**

## Data Availability

All data generated or analysed during this study are included in this published article and its supplementary information files.
