# Supplementary material for: GRADE-ADOLOPMENT of hyperthyroidism treatment guidelines for a Pakistani context
Source: BMC Endocr Disord. 2024 Mar 21;24:41. doi: 10.1186/s12902-023-01493-1 (PMC10956339; doi:10.1186/s12902-023-01493-1)
Supplement: Supplementary file 1 — Additional file 1. [file 12902_2023_1493_MOESM1_ESM.docx]

**Supplementary Material**

1. *Best Evidence Review Process*
2. *Search Strategies for Recommendations that Underwent Adaptations*
3. *Focus Group Discussion Questionnaire*
4. *Supplementary Table 1: Criteria evaluated in the Evidence to Decision (ETD) tables*
5. *Supplementary Table 2: Dummy Evidence to Decision (ETD) table*
6. *Supplementary Table 3: Table of Recommendations for the Management of Hyperthyroidism*
7. *Supplementary Table 4: List of all Excluded Recommendations*

**Best-Evidence Review**

A best-evidence review was conducted to source research evidence that would help a recommendation be assessed across all 12 criteria. The best-evidence review was conducted separately for each of the 12 criteria and included a mini-systematic review and review of supporting evidence.

- *Mini-Systematic Review*: A mini-systematic review follows the same general protocol as a full systematic review, but applies arbitrary selection criteria (such as geographical region of publication) or limits the number of databases searched ^(1)^. In our mini-systematic review, PubMed and Google Scholar were queried using a search string designed using keywords from the recommendation in question. To maintain a local focus, only articles reporting data relevant to Pakistan were selected.

Two members of CCBP staff independently screened the titles and abstracts of articles sourced from PubMed and Google, with only those reporting relevant information specific to Pakistan undergoing a full-text review to finalize inclusion. As the source guideline itself was produced based on a systematic review process, careful full-text review of the bibliography within the source document was conducted. Two members of the CCBP staff extracted appropriate evidence from the final list of articles included.

- *Supporting Evidence*: Information pertaining to the cost of different investigations and treatments, as well as the availability of diagnostic and management facilities, was sourced from a selection of local hospitals, healthcare facilities, and pharmacies via telephonic query and their websites.

**Search Strategies for Recommendations that Underwent Adaptation**

Recommendation 8:

((Graves Disease[MeSH Terms]) OR (Hyperthyroidism[MeSH Terms])) AND ((Radioisotopes[MeSH Terms]) OR (RAI OR Radioiodine OR I-131)) AND ((Hypothyroidism[MeSH Terms]) OR (Hypothyroid OR Hypothyroidism Therapy)) AND ((Dose-Response Relationship Radiation[MeSH Terms]) OR (RAI Dose OR Radioactive Iodine Dose OR I-131 Dose OR Graves Disease Treatment))

Recommendation 11:

((Graves Disease[MeSH Terms]) OR (Hyperthyroidism[MeSH Terms])) AND ((Radioisotopes[MeSH Terms]) OR (Radioactive Iodine[MeSH Terms] OR RAI OR Radioiodine OR I-131)) AND ((Thyroxine[MeSH Terms] OR T4 OR Free T4 OR FT4) OR (Triiodothyronine[MeSH Terms] OR T3 OR Total T3 OR TT3) OR (Thyrotropin[MeSH Terms] OR TSH)) AND (Follow-Up Studies[MeSH Terms])

Recommendation 18:

((Methimazole[MeSH Terms] OR Propylthiouracil[MeSH Terms]) OR (Antithyroid Agents[MeSH Terms] OR ATD OR Antithyroid Drugs OR Antithyroid Medications)) AND ((Liver Function Tests[MeSH Terms]) OR Liver Function OR Hepatocellular Integrity) AND ((Pruritus[MeSH Terms] OR Rash OR Jaundice OR Joint Pain OR Abdominal Pain OR Bloating OR Anorexia OR Nausea OR Fatigue) OR (Symptoms OR Adverse Effects))

Recommendation 24:

((Graves Disease[MeSH Terms]) OR (Hyperthyroidism[MeSH Terms])) AND (Thyroidectomy[MeSH Terms]) AND ((Thyroid Hormones[MeSH Terms]) OR (Antithyroid Agents[MeSH Terms] OR ATD OR Antithyroid Drugs OR Propylthiouracil OR Methimazole OR Methylthiouracil)) AND (Beta Blockers OR Propranolol OR Atenolol OR Metoprolol OR Nadolol) AND ((Potassium Iodide[MeSH Terms]) OR (Iodine OR KI OR Lugol's Solution)) AND (Preoperative Care[MeSH Terms])

**Focus Group Discussion Questionnaire**

**Participant Information:**

Name (optional):

Years of experience in guideline development:

**Section 1: Challenges Faced**

***1.1 Understanding the GRADE-ADOLOPMENT Methodology***

• Can you describe any challenges you faced in understanding the GRADE-ADOLOPMENT methodology when you first encountered it?

**Answer:**

• Did you or your team experience difficulties in interpreting or applying GRADE-ADOLOPMENT concepts? If so, please elaborate.

**Answer:**

***1.2 Identifying and Assessing Evidence***

• What challenges did you encounter when identifying and assessing relevant evidence to inform your guideline development using GRADE-ADOLOPMENT?

**Answer:**

• Were there any specific issues related to the quality of evidence or the inclusion/exclusion criteria that you found difficult?

**Answer:**

***1.3 Incorporating Patient and Stakeholder Input***

• Were there any difficulties in reconciling conflicting perspectives or balancing different stakeholder interests?

**Answer:**

***1.4 Balancing Benefits and Harms***

• Discuss any challenges in weighing the benefits and harms of interventions within the GRADE-ADOLOPMENT framework.

**Answer:**

• Did you encounter difficulties in reaching consensus among team members regarding the balance of benefits and harms?

**Answer:**

***1.5 Grading Recommendations***

• What challenges arose when grading the strength of recommendations using GRADE-ADOLOPMENT criteria?

**Answer:**

• Were there disagreements or uncertainties in your team regarding the final recommendation grades?

**Answer:**

***1.6 Resource***

**•** Did you face any challenges or obstacles due to resource limitations, such as limited access to local data, insufficient relevant studies, or financial constraints?

**Answer:**

**Section 2: Suggestions for Overcoming Challenges**

***2.1 Understanding the Methodology***

• What strategies or resources do you think could help individuals and teams better understand the GRADE-ADOLOPMENT methodology?

**Answer:**

***2.2 Evidence Identification and Assessment***

• Can you suggest ways to streamline the process of identifying and assessing evidence within GRADE-ADOLOPMENT?

**Answer:**

***2.3 Incorporating Patient and Stakeholder Input***

• How can guideline developers better engage patients and stakeholders effectively, and what strategies can help address challenges in this area?

**Answer:**

***2.4 Balancing Benefits and Harms***

• Do you have any recommendations for improving the consensus-building process when balancing benefits and harms within GRADE-ADOLOPMENT?

**Answer:**

***2.5 Grading Recommendations***

• What tools or approaches could assist in simplifying the grading of recommendations under GRADE-ADOLOPMENT?

**Answer:**

***2.6 Resource***

• What are some of the ways in which resource limitations can be overcome?

**Answer:**

| **Supplementary Table 1: Criteria evaluated in the Evidence to Decision (ETD) tables** | | |
| --- | --- | --- |
| **Criteria** | **Description** | **Interpretation** |
| **Problem** | The magnitude of a problem, as measured by its prevalence and severity in a local context | The more serious or urgent a problem is, the more likely that the option that better addresses the problem receives a strong recommendation. |
| **Desirable Effects** | The magnitude of desirable effects is judged by considering the importance of the outcome and the size of the desirable effects (likelihood of experiencing a benefit or degree of benefits an individual experiences). | An option with greater desirable effects is more likely to gain a strong recommendation. |
| **Undesirable Effects** | The magnitude of undesirable effects is similarly judged by considering the importance of the outcome and the size of the undesirable effects. | An option with fewer undesirable effects is more likely to gain a strong recommendation. |
| **Certainty of Evidence** | Determined by likelihood that the research provides valid evidence regarding the effect of the option on all critical outcomes. | Evidence with higher certainty lends to a strong recommendation. |
| **Values** | Magnitude of value is judged by the variability or uncertainty of weightage placed upon the outcome by individuals. | Less variability/uncertainty of value leads to a strong recommendation. |
| **Balance of Effects** | The balance of effects is judged by considering the value individuals place upon the main outcomes, the degree of desirable and undesirable effects, and the certainty of those estimates. | The overall balance of effects can be judged as either favoring the intervention or comparison. |
| **Resources Required** | An estimate of the cost of the difference in resource use between the intervention and comparison. | An option with large savings is more likely to receive a strong recommendation. |
| **Certainty of Evidence of Required Resources** | It is determined by the likelihood that the research provides valid evidence of cost differences between the intervention and comparison. | Evidence with higher certainty lends to a strong recommendation. |
| **Cost-Effectiveness** | Determines cost-effectiveness by considering uncertainty about or variability in costs or net benefit, sensitivity analyses, and the reliability and applicability of the economic evaluation. | An option that is more cost-effective is more likely to receive a strong recommendation |
| **Equity** | Likelihood of differences in the relative effectiveness of the intervention for disadvantaged subgroups that influence the absolute effectiveness of the intervention. | An option with a greater likelihood to favor equity is more likely to receive a strong recommendation. |
| **Acceptability** | Likelihood of key stakeholders to accept the distribution of benefits, harms, costs, and ethical concerns associated with the intervention, over an extended period. | An option more acceptable to most stakeholders is more likely to receive a strong recommendation. |
| **Feasibility** | Practicality of sustained use of the intervention. | An option more feasible to most stakeholders is more likely to receive a strong recommendation. |

| **Supplementary Table 2: Dummy Evidence to Decision (ETD) table** | | | |
| --- | --- | --- | --- |
| **Question**: Should *Intervention/Suggested Change* be favored over *Comparison/Current Standard of Practice*? | | | |
| **Criteria** | **Research Evidence** | **Additional Considerations** | **Judgment** |
| **Problem:** Is the problem a priority? |  |  | - No - Probably No - Probably Yes - Yes - Varies - Don’t Know |
| **Desirable Effects:** How substantial are the desirable anticipated effects? |  |  | - Trivial - Small - Moderate - Large - Varies - Don’t Know |
| **Undesirable Effects**: How substantial are the undesirable anticipated effects? |  |  | - Large - Moderate - Small - Trivial - Varies - Don’t Know |
| **Certainty of Evidence**: What is the overall certainty of the evidence of effects? |  |  | - Very Low - Low - Moderate - High - No Included Studies |
| **Value**: Is there important uncertainty about or variability in how much people value the main outcomes? |  |  | - Important Uncertainty or Variability - Possible Uncertainty or Variability - Probably No Important Uncertainty or Variability - No Important Variability or Uncertainty |
| **Balance of Effects**: Does the balance between desirable and undesirable effects favor the intervention or the comparison? |  |  | - Favors Comparison - Probably Favors the Comparison - Does Not Favor Either the Intervention or Comparison - Probably Favors the Intervention - Favors Intervention - Varies - Don’t Know |
| **Resources Required**: How large are the resource requirements (costs)? |  |  | - Large Costs - Moderate Costs - Negligible Costs or Savings - Moderate Savings - Large Savings - Varies - Don’t Know |
| **Certainty of Evidence of Required Resources**: What is the certainty of the evidence of resource requirements (costs)? |  |  | - Very Low - Low - Moderate - High - No Included Studies |
| **Cost-Effectiveness**: Does the cost-effectiveness of the intervention favor the intervention or the comparison? |  |  | - Favors Comparison - Probably Favors the Comparison - Does Not Favor Either the Intervention or Comparison - Probably Favors the Intervention - Favors Intervention - Varies - No Included Studies |
| **Equity**: What would be the impact on health equity? |  |  | - Reduced - Probably Reduced - Probably No Impact - Probably Increased - Increased - Varies - Don’t Know |
| **Acceptability**: Is the intervention acceptable to key stakeholders? |  |  | - No - Probably No - Probably Yes - Yes - Varies - Don’t Know |
| **Feasibility**: Is the intervention feasible to implement? |  |  | - No - Probably No - Probably Yes - Yes - Varies - Don’t Know |
| **Overall Recommendations**   - Strong Recommendation Against the Intervention - Conditional Recommendation Against the Intervention - Conditional Recommendation for Either the Intervention or the Comparison - Conditional Recommendation for the Intervention - Strong Recommendation for the Intervention | | | |

| **Supplementary Table 3: Table of Recommendations** | | | | |
| --- | --- | --- | --- | --- |
| **S No.** | **Recommendations** | **To Adopt** | **To Adapt/Modify** | **To Remove/Exclude** |
| **1** | [B3] Determination of etiology  **RECOMMENDATION 1**  The etiology of thyrotoxicosis should be determined. If the diagnosis is not apparent based on the clinical presentation and initial biochemical evaluation, diagnostic testing is indicated and can include, depending on available expertise and resources, (1) measurement of TRAb, (2) determination of the radioactive iodine uptake (RAIU), or (3) measurement of thyroidal blood ﬂow on ultrasonography. A 123I or 99mTc pertechnetate scan should be obtained when the clinical presentation suggests a TA or TMNG.  **Strong recommendation, moderate-quality evidence.** | **X** |  |  |
| **2** | [B4] Symptomatic management  **RECOMMENDATION 2**  Beta-adrenergic blockade is recommended in all patients with symptomatic thyrotoxicosis, especially elderly patients and thyrotoxic patients with resting heart rates in excess of 90 beats per minute or coexistent cardiovascular disease.  **Strong recommendation, moderate-quality evidence.** | **X** |  |  |
| **3** | [C] How should overt hyperthyroidism due to GD be managed?  **RECOMMENDATION 3** Patients with overt Graves’ hyperthyroidism should be treated with any of the following modalities: RAI therapy, ATDs, or thyroidectomy.  **Strong recommendation, moderate-quality evidence.** | **X** |  |  |
| **4** | [D] If RAI therapy is chosen, how should it be accomplished?  [D1] Preparation of patients with GD for RAI therapy  **RECOMMENDATION 4**  Because RAI treatment of GD can cause a transient exacerbation of hyperthyroidism, b-adrenergic blockade should be considered even in asymptomatic patients who are at increased risk for complications due to worsening of hyperthyroidism (i.e., elderly patients and patients with comorbidities).  **Weak recommendation, low-quality evidence.** | **X** |  |  |
| **5** | **RECOMMENDATION 5**  In addition to b-adrenergic blockade (see Recommendations 2 and 4), pre-treatment with MMI prior to RAI therapy for GD should be considered in patients who are at increased risk for complications due to worsening of hyperthyroidism. MMI should be discontinued 2–3 days prior to RAI.  **Weak recommendation, moderate-quality evidence.** | **X** |  |  |
| **6** | **RECOMMENDATION 6**  In patients who are at increased risk for complications due to worsening of hyperthyroidism, resuming MMI 3–7 days after RAI administration should be considered.  **Weak recommendation, low-quality evidence.** |  |  | **X** |
| **7** | **RECOMMENDATION 7**  Medical therapy of any comorbid conditions should be optimized prior to RAI therapy.  **Strong recommendation, low-quality evidence.** |  |  | **X** |
| **8** | [D2] Administration of RAI in the treatment of GD  **RECOMMENDATION 8**  Sufﬁcient activity of RAI should be administered in a single application, typically a mean dose of 10–15mCi (370–555MBq), to render the patient with GD hypothyroid.  **Strong recommendation, moderate-quality evidence.** |  | **X** |  |
| **9** | **RECOMMENDATION 9**  A pregnancy test should be obtained within 48 hours prior to treatment in any woman with childbearing potential who is to be treated with RAI. The treating physician should obtain this test and verify a negative result prior to administering RAI.  **Strong recommendation, low-quality evidence.** |  |  | **X** |
| **10** | **RECOMMENDATION 10**  The physician administering RAI should provide written advice concerning radiation safety precautions following treatment. If the precautions cannot be followed, alternative therapy should be selected.  **Strong recommendation, low-quality evidence.** |  |  | **X** |
| **11** | [D3] Patient follow-up after RAI therapy for GD  **RECOMMENDATION 11**  Follow-up within the ﬁrst 1–2 months after RAI therapy for GD should include an assessment of free T4, total T3, and TSH. Biochemical monitoring should be continued at 4- to 6-week intervals for 6 months, or until the patient becomes hypothyroid and is stable on thyroid hormone replacement.  **Strong recommendation, low-quality evidence.** |  | **X** |  |
| **12** | [D4] Treatment of persistent Graves’ hyperthyroidism following RAI therapy  **RECOMMENDATION 12**  When hyperthyroidism due to GD persists after 6 months following RAI therapy, retreatment with RAI is suggested. In selected patients with minimal response 3 months after therapy additional RAI may be considered.  **Weak recommendation, low-quality evidence.** | **X** |  |  |
| **13** | [E1] Initiation of ATD therapy for the treatment of GD  **RECOMMENDATION 13**  MMI should be used in virtually every patient who chooses ATD therapy for GD, except during the ﬁrst trimester of pregnancy when PTU is preferred, in the treatment of thyroid storm, and in patients with minor reactions to MMI who refuse RAI therapy or surgery.  **Strong recommendation, moderate-quality evidence.** | **X** |  |  |
| **14** | **RECOMMENDATION 14**  Patients should be informed of side effects of ATDs and the necessity of informing the physician promptly if they should develop pruritic rash, jaundice, acolic stools or dark urine, arthralgias, abdominal pain, nausea, fatigue, fever, or pharyngitis. Preferably, this information should be in writing. Before starting ATDs and at each subsequent visit, the patient shoulda be alerted to stop the medication immediately and call their physician if there are symptoms suggestive of agranulocytosis or hepatic injury.  **Strong recommendation, low-quality evidence.** | **X** |  |  |
| **15** | **RECOMMENDATION 15**  Prior to initiating ATD therapy for GD, we suggest that patients have a baseline complete blood count, including white blood cell (WBC) count with differential, and a liver proﬁle including bilirubin and transaminases.  **Weak recommendation, low-quality evidence.** | **X** |  |  |
| **16** | **RECOMMENDATION 16**  A differential WBC count should be obtained during febrile illness and at the onset of pharyngitis in all patients taking antithyroid medication.  **Strong recommendation, low-quality evidence.** | **X** |  |  |
| **17** | **RECOMMENDATION 17**  There is insufﬁcient evidence to recommend for or against routine monitoring of WBC counts in patients taking ATDs.  **No recommendation; insufﬁcient evidence to assess beneﬁts and risks.** |  |  | **X** |
| **18** | **RECOMMENDATION 18**  Liver function and hepatocellular integrity should be assessed in patients taking MMI or PTU who experience pruritic rash, jaundice, light-colored stool or dark urine, joint pain, abdominal pain or bloating, anorexia, nausea, or fatigue.  **Strong recommendation, low-quality evidence.** |  | **X** |  |
| **19** | **RECOMMENDATION 19**  There is insufﬁcient information to recommend for or against routine monitoring of liver function tests in patients taking ATDs.  **No recommendation; insufﬁcient evidence to assess beneﬁts and risks.** |  |  | **X** |
| **20** | [E7] Management of allergic reactions  **RECOMMENDATION 20**  Minor cutaneous reactions may be managed with concurrent antihistamine therapy without stopping the ATD. Persistent symptomatic minor side effects of antithyroid medication should be managed by cessation of the medication and changing to RAI or surgery, or switching to the other ATD when RAI or surgery are not options. In the case of a serious allergic reaction, prescribing the alternative drug is not recommended.  **Strong recommendation, low-quality evidence.** | **X** |  |  |
| **21** | [E8] Duration of ATD therapy for GD  **RECOMMENDATION 21**  Measurement of TRAb levels prior to stopping ATD therapy is suggested because it aids in predicting which patients can be weaned from the medication, with normal levels indicating greater chance for remission.  **Strong recommendation, moderate-quality evidence.** | **X** |  |  |
| **22** | **RECOMMENDATION 22**  If MMI is chosen as the primary therapy for GD, the medication should be continued for approximately 12–18 months, then discontinued if the TSH and TRAb levels are normal at that time.  **Strong recommendation, high-quality evidence.** | **X** |  |  |
| **23** | **RECOMMENDATION 23**  If a patient with GD becomes hyperthyroid after completing a course of MMI, consideration should be given to treatment with RAI or thyroidectomy. Continued low-dose MMI treatment for longer than 12–18 months may be considered in patients not in remission who prefer this approach.  **Weak recommendation, low-quality evidence.** | **X** |  |  |
| **24** | [F] If thyroidectomy is chosen for treatment of GD, how should it be accomplished?  [F1] Preparation of patients with GD for thyroidectomy  **RECOMMENDATION 24**  If surgery is chosen as treatment for GD, patients should be rendered euthyroid prior to the procedure with ATD pre-treatment, with or without b-adrenergic blockade. A KI-containing preparation should be given in the immediate preoperative period.  **Strong recommendation, low-quality evidence.** |  | **X** |  |
| **25** | **RECOMMENDATION 25**  Calcium and 25-hydroxy vitamin D should be assessed preoperatively and repleted if necessary or given prophylactically. Calcitriol supplementation should be considered preoperatively in patients at increased risk for transient or permanent hypoparathyroidism.  **Strong recommendation, low-quality evidence.** | **X** |  |  |
| **26** | **RECOMMENDATION 26**  In exceptional circumstances, when it is not possible to render a patient with GD euthyroid prior to thyroidectomy, the need for thyroidectomy is urgent, or when the patient is allergic to ATDs, the patient should be adequately treated with b-adrenergic blockade, KI, glucocorticoids, and potentially cholestyramine in the immediate preoperative period. The surgeon and anesthesiologist should have experience in this situation.  **Strong recommendation, low-quality evidence.** | **X** |  |  |
| **27** | [F2] The surgical procedure and choice of surgeon  **RECOMMENDATION 27**  If surgery is chosen as the primary therapy for GD, near total or total thyroidectomy is the procedure of choice.  **Strong recommendation, moderate-quality evidence.** | **X** |  |  |
| **28** | **RECOMMENDATION 28**  If surgery is chosen as the primary therapy for GD, the patient should be referred to a high-volume thyroid surgeon.  **Strong recommendation, moderate-quality evidence.** | **X** |  |  |
| **29** | [F3] Postoperative care  **RECOMMENDATION 29**  Following thyroidectomy for GD, alternative strategies may be undertaken for management of calcium levels: serum calcium with or without intact parathyroid hormone (iPTH) levels can be measured, and oral calcium and calcitriol supplementation administered based on these results, or prophylactic calcium with or without calcitriol prescribed empirically.  **Weak recommendation, low-quality evidence.** |  |  | **X** |
| **30** | **RECOMMENDATION 30**  ATD should be stopped at the time of thyroidectomy for GD, and b-adrenergic blockers should be weaned following surgery.  **Strong recommendation, low-quality evidence.** |  |  | **X** |
| **31** | **RECOMMENDATION 31**  Following thyroidectomy for GD, L-thyroxine should be started at a daily dose appropriate for the patient’s weight (0.8lg/lb or 1.6lg/kg), with elderly patients needing somewhat less, and serum TSH measured 6–8 weeks postoperatively.  **Strong recommendation, low-quality evidence.** |  |  | **X** |
| **32** | **RECOMMENDATION 32**  Communication among different members of the multidisciplinary team is essential, particularly during transitions of care in the pre- and postoperative settings.  **Strong recommendation, low-quality evidence.** |  |  | **X** |
| **33** | [G] How should thyroid nodules be managed in patients with GD?  **RECOMMENDATION 33**  If a thyroid nodule is discovered in a patient with GD, the nodule should be evaluated and managed according to recently published guidelines regarding thyroid nodules in euthyroid individuals.  **Strong recommendation, moderate-quality evidence.** |  |  | **X** |
| **34** | [H] How should thyroid storm be managed?  **RECOMMENDATION 34** The diagnosis of thyroid storm should be made clinically in a severely thyrotoxic patient with evidence of systemic decompensation. Adjunctive use of a sensitive diagnostic system should be considered. Patients with a Burch-Wartofsky Point Scale (BWPS) of ≥45 or Japanese Thyroid Association (JTA) categories of thyroid storm 1(TS1) or thyroid storm 2 (TS2) with evidence of systemic decompensation require aggressive therapy. The decision to use aggressive therapy in patients with a BWPS of 25–44 should be based on clinical judgment.  Strong recommendation, moderate-quality evidence. | **X** |  |  |
| **35** | **RECOMMENDATION 35**  A multimodality treatment approach to patients with thyroid storm should be used, including b-adrenergic blockade, ATD therapy, inorganic iodide, corticosteroid therapy, cooling with acetaminophen and cooling blankets, volume resuscitation, nutritional support, and respiratory care and monitoring in an intensive care unit, as appropriate for an individual patient.  **Strong recommendation, low-quality evidence.** | **X** |  |  |
| **36** | **RECOMMENDATION 36**  Potassium iodide may be of beneﬁt in select patients with hyperthyroidism due to GD, those who have adverse reactions to ATDs, and those who have a contraindication or aversion to RAI therapy (or aversion to repeat RAI therapy) or surgery. Treatment may be more suitable for patients with mild hyperthyroidism or a prior history of RAI therapy.  **No recommendation: insufﬁcient evidence to assess beneﬁts or risks.** |  |  | **X** |
| **37** | **[J] How should overt hyperthyroidism due to TMNG or TA be managed?**  **RECOMMENDATION 37**  We suggest that patients with overtly TMNG or TA be treated with RAI therapy or thyroidectomy. On occasion, long-term, low-dose treatment with MMI may be appropriate.  **Weak recommendation, moderate-quality evidence.** | **X** |  |  |
| **38** | **[K] If RAI therapy is chosen as treatment for TMNG or TA, how should it be accomplished?**  **[K1] Preparation of patients with TMNG or TA for RAI therapy**  **RECOMMENDATION 38**  Because RAI treatment of TMNG or TA can cause a transient exacerbation of hyperthyroidism, b-adrenergic blockade should be considered even in asymptomatic patients who are at increased risk for complications due to worsening of hyperthyroidism (i.e., elderly patients and patients with comorbidities)  **Weak recommendation, low-quality evidence.** |  |  | **X** |
| **39** | **RECOMMENDATION 39**  In addition to b-adrenergic blockade (see Recommendations 2 and 38) pre-treatment with MMI prior to RAI therapy for TMNG or TA should be considered in patients who are at increased risk for complications due to worsening of hyperthyroidism, including the elderly and those with cardiovascular disease or severe hyperthyroidism.  **Weak recommendation, low-quality evidence.** |  |  | **X** |
| **40** | **RECOMMENDATION 40**  In patients who are at increased risk for complications due to worsening of hyperthyroidism, resuming ATDs 3–7 days after RAI administration should be considered.  **Weak recommendation, low-quality evidence.** |  |  | **X** |
| **41** | [K2] Evaluation of thyroid nodules before RAI therapy  **RECOMMENDATION 41**  Nonfunctioning nodules on radionuclide scintigraphy or nodules with suspicious ultrasound characteristics should be managed according to published guidelines regarding thyroid nodules in euthyroid individuals.  **Strong recommendation, moderate-quality evidence.** |  |  | **X** |
| **42** | [K2] Evaluation of thyroid nodules before RAI therapy  **RECOMMENDATION 41**  Nonfunctioning nodules on radionuclide scintigraphy or nodules with suspicious ultrasound characteristics should be managed according to published guidelines regarding thyroid nodules in euthyroid individuals.  **Strong recommendation, moderate-quality evidence.** |  |  | **X** |
| **43** | **RECOMMENDATION 43**  Sufﬁcient activity of RAI should be administered in a single application to alleviate hyperthyroidism in patients with TA.  **Strong recommendation, moderate-quality evidence.** | **X** |  |  |
| **44** | **RECOMMENDATION 44**  Follow-up within the ﬁrst 1–2 months after RAI therapy for TMNG or TA should include an assessment of free T4, total T3, and TSH. Biochemical monitoring should be continued at 4- to 6-week intervals for 6 months, or until the patient becomes hypothyroid and is stable on thyroid hormone replacement.  **Strong recommendation, low-quality evidence.** |  |  | **X** |
| **45** | [K5] Treatment of persistent or recurrent hyperthyroidism following RAI therapy for TMNG or TA  **RECOMMENDATION 45**  If hyperthyroidism persists beyond 6 months following RAI therapy for TMNG or TA, retreatment with RAI is suggested. In selected patients with minimal response 3 months after therapy additional RAI may be considered.  **Weak recommendation, low-quality evidence.** | **X** |  |  |
| **46** | [L] If surgery is chosen, how should it be accomplished?  [L1] Preparation of patients with TMNG or TA for surgery  **RECOMMENDATION 46**  If surgery is chosen as treatment for TMNG or TA, patients with overt hyperthyroidism should be rendered euthyroid prior to the procedure with MMI pre-treatment, with or without b-adrenergic blockade. Preoperative iodine should not be used in this setting.  **Strong recommendation, low-quality evidence.** |  |  | **X** |
| **47** | [L2] The surgical procedure and choice of surgeon  **RECOMMENDATION 47**  If surgery is chosen as treatment for TMNG, near-total or total thyroidectomy should be performed.  **Strong recommendation, moderate-quality evidence.** | **X** |  |  |
| **48** | **RECOMMENDATION 48**  Surgery for TMNG should be performed by a high-volume thyroid surgeon.  **Strong recommendation, moderate-quality evidence.** | **X** |  |  |
| **49** | **RECOMMENDATION 49**  If surgery is chosen as the treatment for TA, a thyroid ultrasound should be done to evaluate the entire thyroid gland. An ipsilateral thyroid lobectomy, or isthmusectomy if the adenoma is in the thyroid isthmus, should be performed for isolated TAs.  **Strong recommendation, moderate-quality evidence.** | **X** |  |  |
| **50** | **RECOMMENDATION 50**  We suggest that surgery for TA be performed by a high-volume surgeon.  **Weak recommendation, moderate-quality evidence.** |  |  | **X** |
| **51** | **[L3] Postoperative care**  **RECOMMENDATION 51**  Following thyroidectomy for TMNG, serum calcium with or without iPTH levels should be measured, and oral calcium and calcitriol supplementation administered based on the results.  **Weak recommendation, low-quality evidence.** | **X** |  |  |
| **52** | **RECOMMENDATION 52**  MMI should be stopped at the time of surgery for TMNG or TA. Beta-adrenergic blockade should be slowly discontinued following surgery.  **Strong recommendation, low-quality evidence.** | **X** |  |  |
| **53** | **RECOMMENDATION 53**  Following thyroidectomy for TMNG, thyroid hormone replacement should be started at a dose appropriate for the patient’s weight (0.8lg/lb or 1.6lg/kg) and age, with elderly patients needing somewhat less. TSH should be measured every 1–2 months until stable, and then annually.  **Strong recommendation, low-quality evidence.** | **X** |  |  |
| **54** | **RECOMMENDATION 54**  Following lobectomy for TA, TSH and estimated free T4 levels should be obtained 4–6 weeks after surgery and thyroid hormone supplementation started if there is a persistent rise in TSH above the reference range.  **Strong recommendation, low-quality evidence.** | **X** |  |  |
| **55** | [L4] Treatment of persistent or recurrent disease following surgery for TMNG or TA  **RECOMMENDATION 55**  RAI therapy should be used for retreatment of persistent or recurrent hyperthyroidism following inadequate surgery for TMNG or TA.  **Strong recommendation, low-quality evidence.** |  |  | **X** |
| **56** | **RECOMMENDATION 56**  Long-term MMI treatment of TMNG or TA might be indicated in some elderly or otherwise ill patients with limited life expectancy, in patients who are not good candidates for surgery or ablative therapy, and in patients who prefer this option.  **Weak recommendation, low-quality evidence.** | **X** |  |  |
| **57** | [N] Is there a role for ethanol or radiofrequency ablation in the management of TA or TMNG?  **RECOMMENDATION 57**  Alternative therapies such as ethanol or radiofrequency ablation of TA and TMNG can be considered in select patients in whom RAI, surgery, and long-term ATD are inappropriate, contraindicated, or refused, and expertise in these procedures is available.  **No recommendation; insufﬁcient evidence to assess beneﬁts and risks.** |  |  | **X** |
| **58** | [O] How should GD be managed in children and adolescents?  **[O1] General approach**  **RECOMMENDATION 58**  Children with GD should be treated with MMI, RAI therapy, or thyroidectomy. RAI therapy should be avoided in very young children (<5 years). RAI therapy in children is acceptable if the activity is >150lCi/g (5.55MBq/g) of thyroid tissue, and for children between 5 and 10 years of age if the calculated RAI administered activity is <10mCi (<370MBq). Thyroidectomy should be chosen when deﬁnitive therapy is required, the child is too young for RAI, and surgery can be performed by a high-volume thyroid surgeon.  **Strong recommendation, moderate-quality evidence.** |  |  | **X** |
| **59** | [P] If ATDs are chosen as initial management of GD in children, how should the therapy be managed?  [P1] Initiation of ATD therapy for the treatment of GD in children  **RECOMMENDATION 59**  MMI should be used in children who are treated with ATD therapy.  **Strong recommendation, moderate-quality evidence.** |  |  | **X** |
| **60** | **RECOMMENDATION 60**  Pediatric patients and their caretakers should be informed of side effects of ATD preferably in writing, and the necessity of stopping the medication immediately and informing their physician if they develop pruritic rash, jaundice, acolic stools or dark urine, arthralgias, abdominal pain, nausea, fatigue, fever, or pharyngitis.  **Strong recommendation, low-quality evidence.** |  |  | **X** |
| **61** | **RECOMMENDATION 61**  Prior to initiating ATD therapy, we suggest that pediatric patients have, as a baseline, complete blood cell count, including WBC count with differential, and a liver proﬁle including bilirubin, transaminases, and alkaline phosphatase.  **Weak recommendation, low-quality evidence.** |  |  | **X** |
| **62** | [P2] Symptomatic management of Graves’ hyperthyroidism in children  **RECOMMENDATION 62**  Beta-adrenergic blockade is recommended for children experiencing symptoms of hyperthyroidism, especially those with heart rates in excess of 100 beats per minute.  **Strong recommendation, low-quality evidence.** |  |  | **X** |
| **63** | **RECOMMENDATION 63**  ATDs should be stopped immediately and WBC counts measured in children who develop fever, arthralgias, mouth sores, pharyngitis, or malaise.  **Strong recommendation, low-quality evidence.** |  |  | **X** |
| **63** | [P4] Monitoring of children taking PTU  **RECOMMENDATION 64**  In general, PTU should not be used in children. But if it is used, the medication should be stopped immediately and liver function and hepatocellular integrity assessed in children who experience anorexia, pruritus, rash, jaundice, light coloured stool or dark urine, joint pain, right upper quadrant pain or abdominal bloating, nausea, or malaise.  **Strong recommendation, low-quality evidence.** |  |  | **X** |
| **65** | [P5] Management of allergic reactions in children taking MMI  **RECOMMENDATION 65**  Persistent minor cutaneous reactions to MMI therapy in children should be managed by concurrent antihistamine treatment or cessation of the medication and changing to therapy with RAI or surgery. In the case of a serious adverse reaction to an ATD, prescribing the other ATD is not recommended.  **Strong recommendation, low-quality evidence.** |  |  | **X** |
| **66** | **P6 Duration of MMI therapy in children with GD**  **RECOMMENDATION 66**  If MMI is chosen as the ﬁrst-line treatment for GD in children, it may be tapered in those children requiring low doses after 1–2 years to determine if a spontaneous remission has occurred, or it may be continued until the child and caretakers are ready to consider deﬁnitive therapy, if needed.  **Strong recommendation, moderate-quality evidence.** |  |  | **X** |
| **67** | **RECOMMENDATION 67**  Pediatric patients with GD who are not in remission following at least 1–2 years of MMI therapy should be considered for treatment with RAI or thyroidectomy. Alternatively, if children are tolerating ATD therapy, ATDs may be used for extended periods. This approach may be especially useful for the child not considered to be a candidate for either surgery or RAI. Individuals on prolonged ATD therapy (>2 years) should be re-evaluated every 6–12 months and when transitioning to adulthood.  **Strong recommendation, low-quality evidence.** |  |  | **X** |
| **68** | **[Q] If RAI is chosen as treatment for GD in children, how should it be accomplished?**  **[Q1] Preparation of pediatric patients with GD for RAI therapy**  **RECOMMENDATION 68**  We suggest that children with GD having total T4 levels of >20lg/dL (260nmol/L) or free T4 >5ng/dL (60pmol/L) who are to receive RAI therapy be pre-treated with MMI and b-adrenergic blockade until total T4 and/or free T4 normalize before proceeding with RAI treatment.  **Weak recommendation, low-quality evidence.** |  |  | **X** |
| **69** | [Q2] Administration of RAI in the treatment of GD in children  **RECOMMENDATION 69**  If RAI therapy is chosen as treatment for GD in children, sufﬁcient RAI should be administered in a single dose to render the patient hypothyroid.  **Strong recommendation, moderate-quality evidence.** |  |  | **X** |
| **70** | [R] If thyroidectomy is chosen as treatment for GD in children, how should it be accomplished?  [R1] Preparation of children with GD for thyroidectomy  **RECOMMENDATION 70** Children with GD undergoing thyroidectomy should be rendered euthyroid with the use of MMI. A KI-containing preparation should be given in the immediate preoperative period.  **Strong recommendation, low-quality evidence.** |  |  | **X** |
| **71** | **RECOMMENDATION 71**  If surgery is chosen as therapy for GD in children, total or near-total thyroidectomy should be performed.  **Strong recommendation, moderate-quality evidence.** |  |  | **X** |
| **72** | **RECOMMENDATION 72**  Thyroidectomy in children should be performed by high volume thyroid surgeons.  **Strong recommendation, moderate-quality evidence.** |  |  | **X** |
| **73** | [S3] When to treat SH  **RECOMMENDATION 73**  When TSH is persistently <0.1mU/L, treatment of SH is recommended in all individuals ‡65 years of age; in patients with cardiac risk factors, heart disease or osteoporosis; in postmenopausal women who are not on estrogens or bisphosphonates; and in individuals with hyperthyroid symptoms.  **Strong recommendation, moderate-quality evidence.** | **X** |  |  |
| **74** | **RECOMMENDATION 74**  When TSH is persistently <0.1mU/L, treatment of SH should be considered in asymptomatic individuals <65 years of age without the risk factors listed in Recommendation 73.  **Weak recommendation, moderate-quality evidence.** | **X** |  |  |
| **75** | **RECOMMENDATION 75**  When TSH is persistently below the lower limit of normal but ≥0.1mU/L, treatment of SH should be considered in individuals ≥65 years of age and in patients with cardiac disease, osteoporosis, or symptoms of hyperthyroidism.  **Weak recommendation, moderate-quality evidence.** | **X** |  |  |
| **76** | **RECOMMENDATION 76**  When TSH is persistently below the lower limit of normal but ≥0.1mU/L, asymptomatic patients under age 65 without cardiac disease or osteoporosis can be observed without further investigation of the etiology of the subnormal TSH or treatment.  **Weak recommendation, low-quality evidence.** | **X** |  |  |
| **77** | [S4] How to treat SH  **RECOMMENDATION 77**  If SH is to be treated, the treatment should be based on the etiology of the thyroid dysfunction and follow the same principles as outlined for the treatment of overt hyperthyroidism.  **Strong recommendation, low-quality evidence.** | **X** |  |  |
| **78** | [T1] Diagnosis of hyperthyroidism in pregnancy  **RECOMMENDATION 78**  The diagnosis of hyperthyroidism in pregnancy should be made using serum TSH values, and either total T4 and T3 with total T4 and T3 reference ranges increasing to 1.5 times above the nonpregnant range by the second and third trimester or free T4 and total T3 estimations with trimester speciﬁc normal reference ranges.  **Strong recommendation, low-quality evidence.** | **X** |  |  |
| **79** | [T2] Management of hyperthyroidism in pregnancy  **RECOMMENDATION 79**  Transient hCG-mediated TSH suppression in early pregnancy should not be treated with ATD therapy.  **Strong recommendation, low-quality evidence.** | **X** |  |  |
| **80** | **RECOMMENDATION 80**  ATD therapy should be used for overt hyperthyroidism due to GD during pregnancy. PTU should be used when ATD therapy is given during the ﬁrst trimester. MMI should be used when ATD therapy is started after the ﬁrst trimester.  **Strong recommendation, low-quality evidence.** | **X** |  |  |
| **81** | **RECOMMENDATION 81**  In women who develop hyperthyroidism during their reproductive age range, the possibility and timing of future pregnancy should be discussed. Because of the risks of the hyperthyroid state on pregnancy and fetal outcome, we suggest that women should postpone pregnancy until they have become euthyroid with therapy.  **Strong recommendation, low-quality evidence.** | **X** |  |  |
| **82** | **RECOMMENDATION 82**  We suggest that women with hyperthyroidism caused by GD who require high doses of ATDs to achieve euthyroidism should be considered for deﬁnitive therapy before they become pregnant.  **Weak recommendation, low-quality evidence.** | **X** |  |  |
| **83** | **RECOMMENDATION 83**  Women with hyperthyroidism caused by GD that is well controlled on MMI and who desire pregnancy have several options:  a. Patients could consider deﬁnitive therapy before they become pregnant.  b. Patients could switch to PTU before trying to conceive.  c. Patients could switch to PTU as soon as pregnancy is diagnosed.  d. Appropriately selected patients could withdraw from ATD therapy as soon as pregnancy is diagnosed. If ATD therapy is withdrawn, thyroid function should be assessed weekly throughout the ﬁrst trimester, then monthly.  **Weak recommendation, low-quality evidence.** | **X** |  |  |
| **84** | **RECOMMENDATION 84**  We suggest that women who are treated with ATD and who may potentially become pregnant should be instructed to perform a pregnancy test within the ﬁrst days after a missed or unusually light menstrual period.  **Weak recommendation, low-quality evidence.** |  |  | **X** |
| **85** | **RECOMMENDATION 85**  We suggest that a woman who tests positive for pregnancy according to recommendation 84 contact the physician responsible for the ATD therapy within 24 hours to discuss future treatment options.  **Weak recommendation, low-quality evidence.** |  |  | **X** |
| **86** | **RECOMMENDATION 86**  We suggest that the physician contacted according to Recommendation 85 evaluate whether ATD withdrawal in the ﬁrst trimester of pregnancy is likely to cause relapse of hyperthyroidism. Evaluation should be based on patient records, especially the severity of GD at time of diagnosis and current disease activity, duration of ATD therapy, current ATD dose requirement, and results of recent thyroid function and TRAb testing. If risk of relapse is considered low, therapy can be withdrawn and followed by weekly thyroid function testing during the ﬁrst trimester.  **Weak recommendation, low-quality evidence.** | **X** |  |  |
| **87** | **RECOMMENDATION 87**  We suggest that women in early pregnancy who have a high risk of recurrent or worsening hyperthyroidism if ATD is withdrawn be shifted from MMI to PTU immediately after diagnosing pregnancy.  **Weak recommendation, low-quality evidence.** | **X** |  |  |
| **88** | **RECOMMENDATION 88**  Women taking PTU during the ﬁrst trimester of pregnancy according to Recommendations 80, 83, or 87 may be switched to MMI at the beginning of the second trimester, or they may continue PTU therapy for the remaining part of pregnancy if ATD is needed.  **No recommendation; insufﬁcient evidence to assess beneﬁts and risks.** | **X** |  |  |
| **89** | **RECOMMENDATION 89**  GD during pregnancy should be treated with the lowest possible dose of ATD needed to keep the mother’s thyroid hormone levels at or slightly above the reference range for total T4 and T3 values in pregnancy (1.5 times above nonpregnant reference ranges in the second and third trimesters), and the TSH below the reference range for pregnancy. Similarly, free T4 levels should be kept at or slightly above the upper limit of the pregnancy trimester reference range for the assay. Thyroid function should be assessed at least monthly, and the ATD dose adjusted, as required.  **Strong recommendation, low-quality evidence.** | **X** |  |  |
| **90** | **RECOMMENDATION 90**  Pregnancy is a relative contraindication to thyroidectomy and should only be used when medical management has been unsuccessful or ATDs cannot be used.  **Strong recommendation, low-quality evidence.** | **X** |  |  |
| **91** | **RECOMMENDATION 91**  When thyroidectomy is necessary for the treatment of hyperthyroidism during pregnancy, the surgery should be performed if possible during the second trimester.  **Strong recommendation, low-quality evidence.** | **X** |  |  |
| **92** | [T3] The role of TRAb level measurement in pregnancy  **RECOMMENDATION 92**  TRAb levels should be measured when the etiology of hyperthyroidism in pregnancy is uncertain.  **Strong recommendation, low-quality evidence.** | **X** |  |  |
| **93** | **RECOMMENDATION 93**  Patients who were treated with RAI or thyroidectomy for GD prior to pregnancy should have TRAb levels measured using a sensitive assay initially during the ﬁrst trimester thyroid function testing and, if levels are elevated, again at 18–22 weeks of gestation.  **Strong recommendation, low-quality evidence.** | **X** |  |  |
| **94** | **RECOMMENDATION 94**  Patients receiving ATD for GD when becoming pregnant or found to have GD during pregnancy should have TRAb levels measured at initial pregnancy visit or at diagnosis using a sensitive assay and, if they are elevated, again at 18–22 weeks of gestation.  **Strong recommendation, low-quality evidence.** | **X** |  |  |
| **95** | **RECOMMENDATION 95**  Patients with elevated TRAb levels at 18–22 weeks of gestation should have TRAb remeasured in late pregnancy (weeks 30–34) to guide decisions regarding neonatal monitoring. An exception to this recommendation is a woman with an intact thyroid who is no longer in need of ATD therapy.  **Strong recommendation, low-quality evidence.** | **X** |  |  |
| **96** | **RECOMMENDATION 96**  In women developing thyrotoxicosis after delivery, selective diagnostic studies should be performed to distinguish postpartum destructive thyroiditis from postpartum GD.  **Strong recommendation, low-quality evidence.** | **X** |  |  |
| **97** | **RECOMMENDATION 97**  In women with symptomatic thyrotoxicosis from postpartum destructive thyroiditis, the judicious use of b-adrenergic blocking agents is recommended**.**  **Strong recommendation, low-quality evidence.** | **X** |  |  |
| **98** | **RECOMMENDATION 98**  In pregnant women diagnosed with hyperthyroidism due to multinodular thyroid autonomy or a solitary TA, special care should be taken not to induce fetal hypothyroidism by ATD therapy.  **Strong recommendation, low-quality evidence.** |  |  | **X** |
| **99** | **RECOMMENDATION 99**  Euthyroidism should be expeditiously achieved and maintained in hyperthyroid patients with GO or risk factors for the development of orbitopathy.  **Strong recommendation, moderate-quality evidence.** | **X** |  |  |
| **100** | **RECOMMENDATION 100**  We recommend clinicians advise patients with GD to stop smoking and refer them to a structured smoking cessation program. As both ﬁrsthand and secondhand smoking increase GO risk, patients exposed to secondhand smoke should be identiﬁed and advised of its negative impact.  **Strong recommendation, moderate-quality evidence.** | **X** |  |  |
| **101** | **RECOMMENDATION 101**  In nonsmoking patients with GD without apparent GO, RAI therapy (without concurrent steroids), ATDs, or thyroidectomy should be considered equally acceptable therapeutic options in regard to risk of GO.  **Strong recommendation, moderate-quality evidence.** | **X** |  |  |
| **102** | **RECOMMENDATION 102**  In smoking patients with GD without apparent GO, RAI therapy, ATDs, or thyroidectomy should be considered equally acceptable therapeutic options in regard to risk of GO.  **Weak recommendation, low-quality evidence.** | **X** |  |  |
| **103** | **RECOMMENDATION 103**  There is insufﬁcient evidence to recommend for or against the use of prophylactic corticosteroids in smokers who receive RAI and have no evidence of GO.  **No recommendation, insufﬁcient evidence.** |  |  | **X** |
| **104** | [U4] Treatment of hyperthyroidism in patients with active GO of mild severity  **RECOMMENDATION 104**  In patients with Graves’ hyperthyroidism who have mild active ophthalmopathy and no risk factors for deterioration of their eye disease, RAI therapy, ATDs, and thyroidectomy should be considered equally acceptable therapeutic options.  **Strong recommendation, moderate-quality evidence.** | **X** |  |  |
| **105** | **RECOMMENDATION 105**  In the absence of any strong contraindication to GC use we suggest considering them for coverage of GD patients with mild active GO who are treated with RAI, even in the absence of risk factors for GO deterioration.  **Weak recommendation, low-quality evidence.** | **X** |  |  |
| **106** | **RECOMMENDATION 106**  In GD patients with mild GO who are treated with RAI we recommend steroid coverage if there are concomitant risk factors for GO deterioration.  **Strong recommendation, moderate-quality evidence.** | **X** |  |  |
| **107** | [U5] Treatment of hyperthyroidism in patients with active and moderate-to-severe or sight-threatening GO  **RECOMMENDATION 107**  In patients with active and moderate-to-severe or sight-threatening GO we recommend against RAI therapy. Surgery or ATDs are preferred treatment options for GD in these patients.  **Strong recommendation, low-quality evidence.** | **X** |  |  |
| **108** | [U6] Treatment of GD in patients with inactive GO  **RECOMMENDATION 108**  In patients with inactive GO we suggest RAI therapy can be administered without steroid coverage. However, in cases of elevated risk for reactivation (high TRAb, CAS≥1 and smokers) that approach might have to be reconsidered.  **Weak recommendation, low-quality evidence.** | **X** |  |  |
| **109** | [V] How should iodine-induced and amiodarone induced thyrotoxicosis be managed?  [V1] Iodine-induced hyperthyroidism  **RECOMMENDATION 109**  Routine administration of ATDs before iodinated contrast media exposure is not recommended for all patients.  **Weak recommendation, low-quality evidence.** | **X** |  |  |
| **110** | **RECOMMENDATION 110**  Beta-adrenergic blocking agents alone or in combination with MMI should be used to treat overt iodine-induced hyperthyroidism.  **Strong recommendation, low-quality evidence.** |  |  | **X** |
| **111** | **[V2] Amiodarone-induced thyrotoxicosis**  **RECOMMENDATION 111**  We suggest monitoring thyroid function tests before and within the ﬁrst 3 months following the initiation of amiodarone therapy, and at 3- to 6-month intervals thereafter.  **Weak recommendation, low-quality evidence.** | **X** |  |  |
| **112** | **RECOMMENDATION 112**  The decision to stop amiodarone in the setting of thyrotoxicosis should be determined on an individual basis in consultation with the treating cardiologist, depending on the clinical manifestations and presence or absence of effective alternative antiarrhythmic therapy.  **Strong recommendation, low-quality evidence.** | **X** |  |  |
| **113** | **RECOMMENDATION 113**  In clinically stable patients with AIT, we suggest measuring thyroid function tests to identify disorders associated with iodine-induced hyperthyroidism (type 1 AIT), speciﬁcally including toxic nodular disease and previously occult GD**.**  **Strong recommendation, low-quality evidence.** | **X** |  |  |
| **114** | **RECOMMENDATION 114**  CBZ/MMI should be used to treat overt thyrotoxicosis in patients with proven underlying autonomous thyroid nodules or GD as the cause of AIT (type 1 disease), and corticosteroids should be used to treat patients with overt amiodarone-induced thyroiditis (type 2 disease).  **Strong recommendation, low-quality evidence.** | **X** |  |  |
| **115** | **RECOMMENDATION 115**  Combined ATD and corticosteroid therapy should be used to treat patients with overt AIT who are too unstable clinically to allow a trial of monotherapy or who fail to respond to single modality therapy, or patients in whom the etiology of thyrotoxicosis cannot be unequivocally determined.  **Strong recommendation, low-quality evidence.** |  |  | **X complicated for PCP**  **REFER** |
| **116** | **RECOMMENDATION 116**  Patients with AIT who are unresponsive to aggressive medical therapy with MMI and corticosteroids should undergo thyroidectomy.  **Strong recommendation, low-quality evidence.** |  |  | **X complicated for PCP**  **REFER** |
| **117** | **RECOMMENDATION 117**  Patients with mild symptomatic subacute thyroiditis should be treated initially with b-adrenergic-blocking drugs and nonsteroidal anti-inﬂammatory agents (NSAIDs). Corticosteroids should be used instead of NSAIDs when patients fail to respond or present initially with moderate to severe pain and/or thyrotoxic symptoms.  **Strong recommendation, low-quality evidence.** | **X** |  |  |
| **118** | **RECOMMENDATION 118**  Patients with symptomatic thyrotoxicosis due to painless thyroiditis should be treated with b-adrenergic-blocking drugs to control symptoms.  **Strong recommendation, low-quality evidence.** | **X** |  |  |
| **119** | [W3] Acute thyroiditis  **RECOMMENDATION 119**  Acute thyroiditis should be treated with antibiotics and surgical drainage as determined by clinical judgement. Beta-blockers may be used to treat symptoms of thyrotoxicosis.  **Strong recommendation, low-quality evidence.** | **X** |  |  |
| **120** | [X] How should other causes of thyrotoxicosis be managed?  **RECOMMENDATION 120**  Patients taking medications known to cause thyrotoxicosis, including interferon (IFN)-a, interleukin-2, tyrosine kinase inhibitors, and lithium, should be monitored clinically and biochemically at 6-month intervals for the development of thyroid dysfunction. Patients who develop thyrotoxicosis should be evaluated to determine etiology and treated accordingly.  **Strong recommendation, low-quality evidence.** |  |  | **X** |
| **121** | [X4] TSH-secreting pituitary tumors  **RECOMMENDATION 121**  The diagnosis of a TSH-secreting pituitary adenomas should be based on an inappropriately normal or elevated serum TSH level associated with elevated free T4 and total T3 concentrations, generally associated with a pituitary tumor on MRI or CT and the absence of a family history or genetic testing consistent with resistance to thyroid hormone.  **Strong recommendation, low-quality evidence.** |  |  | **X complicated for PCP**  **REFER** |
| **122** | **RECOMMENDATION 122**  Patients with TSH-secreting pituitary adenomas should undergo surgery performed by an experienced pituitary surgeon.  **Strong recommendation, low-quality evidence.** |  |  | **X** |
| **123** | [X5] Struma ovarii  **RECOMMENDATION 123**  Patients with struma ovarii should be treated initially with surgical resection following preoperative normalization of thyroid hormones.  **Strong recommendation, low-quality evidence.** |  |  | **X** |
| **124** | [X6] Choriocarcinoma  **RECOMMENDATION 124**  Treatment of hyperthyroidism due to choriocarcinoma should include both MMI and treatment directed against the primary tumor.  **Strong recommendation, low-quality evidence.** |  |  | **X** |

| **Supplementary Table 4: List of all Excluded Recommendations** | | |
| --- | --- | --- |
|  | **S No.** | **Recommendations** |
| **1** | **6** | **RECOMMENDATION 6**  In patients who are at increased risk for complications due to worsening of hyperthyroidism, resuming MMI 3–7 days after RAI administration should be considered.  **Weak recommendation, low-quality evidence.** |
| **2** | **7** | **RECOMMENDATION 7**  Medical therapy of any comorbid conditions should be optimized prior to RAI therapy.  **Strong recommendation, low-quality evidence.** |
| **3** | **9** | **RECOMMENDATION 9**  A pregnancy test should be obtained within 48 hours prior to treatment in any woman with childbearing potential who is to be treated with RAI. The treating physician should obtain this test and verify a negative result prior to administering RAI.  **Strong recommendation, low-quality evidence.** |
| **4** | **10** | **RECOMMENDATION 10**  The physician administering RAI should provide written advice concerning radiation safety precautions following treatment. If the precautions cannot be followed, alternative therapy should be selected.  **Strong recommendation, low-quality evidence.** |
| **5** | **17** | **RECOMMENDATION 17**  There is insufﬁcient evidence to recommend for or against routine monitoring of WBC counts in patients taking ATDs.  **No recommendation; insufﬁcient evidence to assess beneﬁts and risks.** |
| **6** | **19** | **RECOMMENDATION 19**  There is insufﬁcient information to recommend for or against routine monitoring of liver function tests in patients taking ATDs.  **No recommendation; insufﬁcient evidence to assess beneﬁts and risks.** |
| **7** | **29** | [F3] Postoperative care  **RECOMMENDATION 29**  Following thyroidectomy for GD, alternative strategies may be undertaken for management of calcium levels: serum calcium with or without intact parathyroid hormone (iPTH) levels can be measured, and oral calcium and calcitriol supplementation administered based on these results, or prophylactic calcium with or without calcitriol prescribed empirically.  **Weak recommendation, low-quality evidence.** |
| **8** | **30** | **RECOMMENDATION 30**  ATD should be stopped at the time of thyroidectomy for GD, and b-adrenergic blockers should be weaned following surgery.  **Strong recommendation, low-quality evidence.** |
| **9** | **31** | **RECOMMENDATION 31**  Following thyroidectomy for GD, L-thyroxine should be started at a daily dose appropriate for the patient’s weight (0.8lg/lb or 1.6lg/kg), with elderly patients needing somewhat less, and serum TSH measured 6–8 weeks postoperatively.  **Strong recommendation, low-quality evidence.** |
| **10** | **32** | **RECOMMENDATION 32**  Communication among different members of the multidisciplinary team is essential, particularly during transitions of care in the pre- and postoperative settings.  **Strong recommendation, low-quality evidence.** |
| **11** | **33** | [G] How should thyroid nodules be managed in patients with GD?  **RECOMMENDATION 33**  If a thyroid nodule is discovered in a patient with GD, the nodule should be evaluated and managed according to recently published guidelines regarding thyroid nodules in euthyroid individuals.  **Strong recommendation, moderate-quality evidence.** |
| **12** | **36** | **RECOMMENDATION 36**  Potassium iodide may be of beneﬁt in select patients with hyperthyroidism due to GD, those who have adverse reactions to ATDs, and those who have a contraindication or aversion to RAI therapy (or aversion to repeat RAI therapy) or surgery. Treatment 13may be more suitable for patients with mild hyperthyroidism or a prior history of RAI therapy.  **No recommendation: insufﬁcient evidence to assess beneﬁts or risks.** |
| **13** | **38** | **[K] If RAI therapy is chosen as treatment for TMNG or TA, how should it be accomplished?**  **[K1] Preparation of patients with TMNG or TA for RAI therapy**  **RECOMMENDATION 38**  Because RAI treatment of TMNG or TA can cause a transient exacerbation of hyperthyroidism, b-adrenergic blockade should be considered even in asymptomatic patients who are at increased risk for complications due to worsening of hyperthyroidism (i.e., elderly patients and patients with comorbidities)  **Weak recommendation, low-quality evidence.** |
| **14** | **39** | **RECOMMENDATION 39**  In addition to b-adrenergic blockade (see Recommendations 2 and 38) pre-treatment with MMI prior to RAI therapy for TMNG or TA should be considered in patients who are at increased risk for complications due to worsening of hyperthyroidism, including the elderly and those with cardiovascular disease or severe hyperthyroidism.  **Weak recommendation, low-quality evidence.** |
| **15** | **40** | **RECOMMENDATION 40**  In patients who are at increased risk for complications due to worsening of hyperthyroidism, resuming ATDs 3–7 days after RAI administration should be considered.  **Weak recommendation, low-quality evidence.** |
| **16** | **41** | [K2] Evaluation of thyroid nodules before RAI therapy  **RECOMMENDATION 41**  Nonfunctioning nodules on radionuclide scintigraphy or nodules with suspicious ultrasound characteristics should be managed according to published guidelines regarding thyroid nodules in euthyroid individuals.  **Strong recommendation, moderate-quality evidence.** |
| **17** | **42** | [K2] Evaluation of thyroid nodules before RAI therapy  **RECOMMENDATION 41**  Nonfunctioning nodules on radionuclide scintigraphy or nodules with suspicious ultrasound characteristics should be managed according to published guidelines regarding thyroid nodules in euthyroid individuals.  **Strong recommendation, moderate-quality evidence.** |
| **18** | **44** | **RECOMMENDATION 44**  Follow-up within the ﬁrst 1–2 months after RAI therapy for TMNG or TA should include an assessment of free T4, total T3, and TSH. Biochemical monitoring should be continued at 4- to 6-week intervals for 6 months, or until the patient becomes hypothyroid and is stable on thyroid hormone replacement.  **Strong recommendation, low-quality evidence.** |
| **19** | **46** | [L] If surgery is chosen, how should it be accomplished?  [L1] Preparation of patients with TMNG or TA for surgery  **RECOMMENDATION 46**  If surgery is chosen as treatment for TMNG or TA, patients with overt hyperthyroidism should be rendered euthyroid prior to the procedure with MMI pre-treatment, with or without b-adrenergic blockade. Preoperative iodine should not be used in this setting.  **Strong recommendation, low-quality evidence.** |
| **20** | **50** | **RECOMMENDATION 50**  We suggest that surgery for TA be performed by a high-volume surgeon.  **Weak recommendation, moderate-quality evidence.** |
| **21** | **55** | [L4] Treatment of persistent or recurrent disease following surgery for TMNG or TA  **RECOMMENDATION 55**  RAI therapy should be used for retreatment of persistent or recurrent hyperthyroidism following inadequate surgery for TMNG or TA.  **Strong recommendation, low-quality evidence.** |
| **22** | **57** | [N] Is there a role for ethanol or radiofrequency ablation in the management of TA or TMNG?  **RECOMMENDATION 57**  Alternative therapies such as ethanol or radiofrequency ablation of TA and TMNG can be considered in select patients in whom RAI, surgery, and long-term ATD are inappropriate, contraindicated, or refused, and expertise in these procedures is available.  **No recommendation; insufﬁcient evidence to assess beneﬁts and risks.** |
| **23** | **58** | [O] How should GD be managed in children and adolescents?  **[O1] General approach**  **RECOMMENDATION 58**  Children with GD should be treated with MMI, RAI therapy, or thyroidectomy. RAI therapy should be avoided in very young children (<5 years). RAI therapy in children is acceptable if the activity is >150lCi/g (5.55MBq/g) of thyroid tissue, and for children between 5 and 10 years of age if the calculated RAI administered activity is <10mCi (<370MBq). Thyroidectomy should be chosen when deﬁnitive therapy is required, the child is too young for RAI, and surgery can be performed by a high-volume thyroid surgeon.  **Strong recommendation, moderate-quality evidence.** |
| **24** | **59** | [P] If ATDs are chosen as initial management of GD in children, how should the therapy be managed?  [P1] Initiation of ATD therapy for the treatment of GD in children  **RECOMMENDATION 59**  MMI should be used in children who are treated with ATD therapy.  **Strong recommendation, moderate-quality evidence.** |
| **25** | **60** | **RECOMMENDATION 60**  2Pediatric patients and their caretakers should be informed of side effects of ATD preferably in writing, and the necessity of stopping the medication immediately and informing their physician if they develop pruritic rash, jaundice, acolic stools or dark urine, arthralgias, abdominal pain, nausea, fatigue, fever, or pharyngitis.  **Strong recommendation, low-quality evidence.** |
| **26** | **61** | **RECOMMENDATION 61**  Prior to initiating ATD therapy, we suggest that pediatric patients have, as a baseline, complete blood cell count, including WBC count with differential, and a liver proﬁle including bilirubin, transaminases, and alkaline phosphatase.  **Weak recommendation, low-quality evidence.** |
| **27** | **62** | [P2] Symptomatic management of Graves’ hyperthyroidism in children  **RECOMMENDATION 62**  Beta-adrenergic blockade is recommended for children experiencing symptoms of hyperthyroidism, especially those with heart rates in excess of 100 beats per minute.  **Strong recommendation, low-quality evidence.** |
| **28** | **63** | **RECOMMENDATION 63**  ATDs should be stopped immediately and WBC counts measured in children who develop fever, arthralgias, mouth sores, pharyngitis, or malaise.  **Strong recommendation, low-quality evidence.** |
| **29** | **63** | [P4] Monitoring of children taking PTU  **RECOMMENDATION 64**  In general, PTU should not be used in children. But if it is used, the medication should be stopped immediately and liver function and hepatocellular integrity assessed in children who experience anorexia, pruritus, rash, jaundice, light coloured stool or dark urine, joint pain, right upper quadrant pain or abdominal bloating, nausea, or malaise.  **Strong recommendation, low-quality evidence.** |
| **30** | **65** | [P5] Management of allergic reactions in children taking MMI  **RECOMMENDATION 65**  Persistent minor cutaneous reactions to MMI therapy in children should be managed by concurrent antihistamine treatment or cessation of the medication and changing to therapy with RAI or surgery. In the case of a serious adverse reaction to an ATD, prescribing the other ATD is not recommended.  **Strong recommendation, low-quality evidence.** |
| **31** | **66** | **P6 Duration of MMI therapy in children with GD**  **RECOMMENDATION 66**  If MMI is chosen as the ﬁrst-line treatment for GD in children, it may be tapered in those children requiring low doses after 1–2 years to determine if a spontaneous remission has occurred, or it may be continued until the child and caretakers are ready to consider deﬁnitive therapy, if needed.  **Strong recommendation, moderate-quality evidence.** |
| **32** | **67** | **RECOMMENDATION 67**  Pediatric patients with GD who are not in remission following at least 1–2 years of MMI therapy should be considered for treatment with RAI or thyroidectomy. Alternatively, if children are tolerating ATD therapy, ATDs may be used for extended periods. This approach may be especially useful for the child not considered to be a candidate for either surgery or RAI. Individuals on prolonged ATD therapy (>2 years) should be re-evaluated every 6–12 months and when transitioning to adulthood.  **Strong recommendation, low-quality evidence.** |
| **33** | **68** | **[Q] If RAI is chosen as treatment for GD in children, how should it be accomplished?**  **[Q1] Preparation of pediatric patients with GD for RAI therapy**  **RECOMMENDATION 68**  We suggest that children with GD having total T4 levels of >20lg/dL (260nmol/L) or free T4 >5ng/dL (60pmol/L) who are to receive RAI therapy be pre-treated with MMI and b-adrenergic blockade until total T4 and/or free T4 normalize before proceeding with RAI treatment.  **Weak recommendation, low-quality evidence.** |
| **34** | **69** | [Q2] Administration of RAI in the treatment of GD in children  **RECOMMENDATION 69**  If RAI therapy is chosen as treatment for GD in children, sufﬁcient RAI should be administered in a single dose to render the patient hypothyroid.  **Strong recommendation, moderate-quality evidence.** |
| **35** | **70** | [R] If thyroidectomy is chosen as treatment for GD in children, how should it be accomplished?  [R1] Preparation of children with GD for thyroidectomy  **RECOMMENDATION 70** Children with GD undergoing thyroidectomy should be rendered euthyroid with the use of MMI. A KI-containing preparation should be given in the immediate preoperative period.  **Strong recommendation, low-quality evidence.** |
| **36** | **71** | **RECOMMENDATION 71**  If surgery is chosen as therapy for GD in children, total or near-total thyroidectomy should be performed.  **Strong recommendation, moderate-quality evidence.** |
| **37** | **72** | **RECOMMENDATION 72**  Thyroidectomy in children should be performed by high volume thyroid surgeons.  **Strong recommendation, moderate-quality evidence.** |
| **38** | **84** | **RECOMMENDATION 84**  We suggest that women who are treated with ATD and who may potentially become pregnant should be instructed to perform a pregnancy test within the ﬁrst days after a missed or unusually light menstrual period.  **Weak recommendation, low-quality evidence.** |
| **39** | **85** | **RECOMMENDATION 85**  We suggest that a woman who tests positive for pregnancy according to recommendation 84 contact the physician responsible for the ATD therapy within 24 hours to discuss future treatment options.  **Weak recommendation, low-quality evidence.** |
| **40** | **98** | **RECOMMENDATION 98**  In pregnant women diagnosed with hyperthyroidism due to multinodular thyroid autonomy or a solitary TA, special care should be taken not to induce fetal hypothyroidism by ATD therapy.  **Strong recommendation, low-quality evidence.** |
| **41** | **103** | **RECOMMENDATION 103**  There is insufﬁcient evidence to recommend for or against the use of prophylactic corticosteroids in smokers who receive RAI and have no evidence of GO.  **No recommendation, insufﬁcient evidence.** |
| **42** | **110** | **RECOMMENDATION 110**  Beta-adrenergic blocking agents alone or in combination with MMI should be used to treat overt iodine-induced hyperthyroidism.  **Strong recommendation, low-quality evidence.** |
| **43** | **115** | **RECOMMENDATION 115**  Combined ATD and corticosteroid therapy should be used to treat patients with overt AIT who are too unstable clinically to allow a trial of monotherapy or who fail to respond to single modality therapy, or patients in whom the etiology of thyrotoxicosis cannot be unequivocally determined.  **Strong recommendation, low-quality evidence.** |
| **44** | **116** | **RECOMMENDATION 116**  Patients with AIT who are unresponsive to aggressive medical therapy with MMI and corticosteroids should undergo thyroidectomy.  **Strong recommendation, low-quality evidence.** |
| **45** | **120** | [X] How should other causes of thyrotoxicosis be managed?  **RECOMMENDATION 120**  Patients taking medications known to cause thyrotoxicosis, including interferon (IFN)-a, interleukin-2, tyrosine kinase inhibitors, and lithium, should be monitored clinically and biochemically at 6-month intervals for the development of thyroid dysfunction. Patients who develop thyrotoxicosis should be evaluated to determine etiology and treated accordingly.  **Strong recommendation, low-quality evidence.** |
| **46** | **121** | [X4] TSH-secreting pituitary tumors  **RECOMMENDATION 121**  The diagnosis of a TSH-secreting pituitary adenomas should be based on an inappropriately normal or elevated serum TSH level associated with elevated free T4 and total T3 concentrations, generally associated with a pituitary tumor on MRI or CT and the absence of a family history or genetic testing consistent with resistance to thyroid hormone.  **Strong recommendation, low-quality evidence.** |
| **47** | **122** | **RECOMMENDATION 122**  Patients with TSH-secreting pituitary adenomas should undergo surgery performed by an experienced pituitary surgeon.  **Strong recommendation, low-quality evidence.** |
| **48** | **123** | [X5] Struma ovarii  **RECOMMENDATION 123**  Patients with struma ovarii should be treated initially with surgical resection following preoperative normalization of thyroid hormones.  **Strong recommendation, low-quality evidence.** |
| **49** | **124** | [X6] Choriocarcinoma  **RECOMMENDATION 124**  Treatment of hyperthyroidism due to choriocarcinoma should include both MMI and treatment directed against the primary tumor.  **Strong recommendation, low-quality evidence.** |
