# Supplementary material for: GRADE-ADOLOPMENT of hyperthyroidism treatment guidelines for a Pakistani context
Source: BMC Endocr Disord. 2024 Mar 21;24:41. doi: 10.1186/s12902-023-01493-1 (PMC10956339; doi:10.1186/s12902-023-01493-1)
Supplement: Supplementary file 2 — Additional file 2. [file 12902_2023_1493_MOESM2_ESM.docx]

|  | |
| --- | --- |
| Supplementary Table 5: Evidence to decision table for recommendation # 11 | |
| Question | |
| **Should TFTs frequency be 1-2months post initial RAI and q4-6weekly afterwards for 6 months vs. no tests or frequency of testing be used for GD patients Post RAI therapy?** | |
| **Population:** | GD patients Post RAI therapy |
| **Intervention:** | TFTs frequency be 1-2months post initial RAI and q4-6weekly afterwards for 6 months |
| **Comparison:** | no tests or frequency of testing |
| **Main outcomes:** |  |
| **Setting:** | Pakistan, South Asia |
| **Perspective:** |  |
| **Background:** |  |
| **Conflict of interests:** |  |

# Assessment

| Problem Is the problem a priority? | |
| --- | --- |
| Judgement | Research evidence |
| ○ No ○ Probably no ○ Probably yes ● Yes ○ Varies ○ Don't know | In Pakistan, the prevalence of hyperthyroidism is 5.1% and is higher in females than males (1).    1. Hussain, A., & Avais, J. (2020). Grave’s Disease: When to Jump for Operative Management. *Archives of Surgical Research*, *1*(4), 12-16. Retrieved from [http://archivessr.com/index.php/asr/article/view/4](http://archivessr.com/index.php/asr/article/view/49)9  --------------------------------------------------------------------------------------  In Pakistan, the prevalence of overt and sub-clinical hyperthyroidism is reported to be 5.1% and 5.8%, respectively. Similarly, the prevalence of overt and sub-clinical hypothyroidism is observed at 4.1 and 5.4%, respectively. It is also perceived that the prevalence of both hyperthyroidism and hypothyroidism (subclinical or overt) is higher in females than males (2).    2. Iqbal, A., et al. (2016). Prevalence and manifestations of thyroidal dysfunction in central Punjab Pakistan. Sci.Int.(Lahore),28(4),3959-3963. Retrieved from http://www.sci-int.com/pdf/636428150898615705.%20Amir%20Iqbal--ZOO--PU--28-6-16--REVIEWED%20by%20Zahid.pdf  ----------------------------------------------------------------------------------------- |
| Desirable Effects How substantial are the desirable anticipated effects? | |
| Judgement | Research evidence |
| ○ Trivial ○ Small ● Moderate ○ Large ○ Varies ○ Don't know | **Evidence from Source Guideline:**    "Most patients respond to RAI therapy with normalization of thyroid function tests and improvement of clinical symptoms within 4–8 weeks. Hypothyroidism may occur from 4 weeks on, with 40% of patients being hypothyroid by 8weeks and >80% by 16 weeks (3). This transition can occur rapidly but more commonly between 2 and 6 months, and the timing of thyroid hormone replacement therapy should be determined by results of thyroid function tests, clinical symptoms, and physical examination".    "2016 American Thyroid Association Guidelines for Diagnosis and Management of Hyperthyroidism and Other Causes of Thyrotoxicosis"    3. Stan MN, Durski JM, Brito JP, Bhagra S, Thapa P, Bahn RS 2013 Cohort study on radioactive iodine-induced hypothyroidism: implications for Graves’ ophthalmopathy and optimal timing for thyroid hormone assessment. Thyroid 23:620–625.  --------------------------------------------------------------------------------------  **Local Evidence:**    Literature search on PubMed found no local studies with evidence for the follow-ups of Thyroid function tests after Radioactive iodine therapy. Our inclusion criteria were adults >18 years of patients with Graves’ Disease in Pakistan/South Asia from the date 1 Jan 2016 till 15 Sept 2021.    There are no direct comparative studies that compare different intervals and assess them on patient-important outcomes.  ---------------------------------------------------------------------------------------- |
| Undesirable Effects How substantial are the undesirable anticipated effects? | |
| Judgement | Research evidence |
| ○ Large ○ Moderate ● Small ○ Trivial ○ Varies ○ Don't know | **Evidence from Source Guideline:**    "Transient hypothyroidism following RAI therapy can rarely occur, with subsequent complete recovery of thyroid function or recurrent hyperthyroidism (4)".  "2016 American Thyroid Association Guidelines for Diagnosis and Management of Hyperthyroidism and Other Causes of Thyrotoxicosis.    4. Uy HL, Reasner CA, Samuels MH 1995 Pattern of recovery of the hypothalamic-pituitary-thyroid axis following radioactive iodine therapy in patients with Graves’ disease. Am J Med 99:173–179.  --------------------------------------------------------------------------------------  **Local Evidence:**    Literature search on PubMed found no local studies found for any adverse effects of radioactive iodine therapy in grave disease patients. Our inclusion criteria were adults >18 years of patients with Graves’ Disease in Pakistan/South Asia from the date 1 Jan 2016 till 15 Sept 2021.    There are no direct comparative studies that compare different intervals and assess them on patient-important outcomes.  ----------------------------------------------------------------------------------------- |
| Certainty of evidence What is the overall certainty of the evidence of effects? | |
| Judgement | Research evidence |
| ○ Very low ● Low ○ Moderate ○ High ○ No included studies | **Evidence from Source Guideline:**    "Most patients respond to RAI therapy with normalization of thyroid function tests and improvement of clinical symptoms within 4–8 weeks. Hypothyroidism may occur from 4 weeks on, with 40% of patients being hypothyroid by 8weeks and >80% by 16 weeks (3). This transition can occur rapidly but more commonly between 2 and 6 months, and the timing of thyroid hormone replacement therapy should be determined by results of thyroid function tests, clinical symptoms, and physical examination".    "2016 American Thyroid Association Guidelines for Diagnosis and Management of Hyperthyroidism and Other Causes of Thyrotoxicosis".    3. Stan MN, Durski JM, Brito JP, Bhagra S, Thapa P, Bahn RS 2013 Cohort study on radioactive iodine-induced hypothyroidism: implications for Graves’ ophthalmopathy and optimal timing for thyroid hormone assessment. Thyroid 23:620–625.  -------------------------------------------------------------------------------------  **Local Evidence:**    Literature search on PubMed found NO studies with the local context. Our inclusion criteria were adults >18 years of patients with Graves’ Disease in Pakistan/South Asia from the date 1 Jan 2016 till 15 Sept 2021.    There are no direct comparative studies that compare different intervals and assess them on patient-important outcomes.  ----------------------------------------------------------------------------------------- |
| Values Is there important uncertainty about or variability in how much people value the main outcomes? | |
| Judgement | Research evidence |
| ○ Important uncertainty or variability ○ Possibly important uncertainty or variability ● Probably no important uncertainty or variability ○ No important uncertainty or variability | **Evidence from Source Guideline:**    No evidence from the source guideline is pertinent to this query in original source guideline (2016 American Thyroid Association Guidelines for Diagnosis and Management of Hyperthyroidism and Other Causes of Thyrotoxicosis).  ---------------------------------------------------------------------------  **Local Evidence:**    Literature search on PubMed found NO studies with the local context. Our inclusion criteria were adults >18 years of patients with Graves’ Disease in Pakistan/South Asia from the date 1 Jan 2016 till 15 Sept 2021.    No patient advocate input is available.    No local evidence is available on patient values.  ----------------------------------------------------------------------------------------- |
| Balance of effects Does the balance between desirable and undesirable effects favor the intervention or the comparison? | |
| Judgement | Research evidence |
| ○ Favors the comparison ○ Probably favors the comparison ○ Does not favor either the intervention or the comparison ● Probably favors the intervention ○ Favors the intervention ○ Varies ○ Don't know | Balance of effects is based on the above desirable vs undesirable effects judgments.    Panel members, please review the above data described in sections 2 and 3, desirable and undesirable effects.  Make your own judgments about the balance of effects. |
| Resources required How large are the resource requirements (costs)? | |
| Judgement | Research evidence |
| ○ Large costs ● Moderate costs ○ Negligible costs and savings ○ Moderate savings ○ Large savings ○ Varies ○ Don't know | As per telephonic research, cost of the TFTs in Pakistan/ South Asia is as following:    **AKUH:**  · Thyroid profile=3850 PKR  · T3=1600 PKR  · T4= 2100 PKR  · Free T4= 2750 PKR  · Free T3= 2700 PKR  · TSH= 1900 PKR    **Essa Lab**  · Thyroid profile=2350 PKR  · T3=1390 PKR  · T4=1390 PKR  · Free T4= 800 PKR  · Free T3= 800 PKR  · TSH= 490 PKR    **DOW**  · Thyroid profile=1600 PKR  · T3=1150 PKR  · T4=1150 PKR  · Free T4= 1900 PKR  · Free T3= 1900 PKR  · TSH= 550 PKR    **Chughtai Lab**  · Thyroid profile=5500 PKR  · T3=1200 PKR  · T4=1200 PKR  · Free T4= 2000 PKR  · Free T3= 2000 PKR  · TSH= 1300 PKR |
| Certainty of evidence of required resources What is the certainty of the evidence of resource requirements (costs)? | |
| Judgement | Research evidence |
| ○ Very low ○ Low ● Moderate ○ High ○ No included studies | As per google search the below links are available for further information and verification for laboratory tests:  · <https://hospitals.aku.edu/pakistan/Pages/Lab-Tests-Directory.aspx>  · <https://www.dressalab.com/>  · <https://www.duhs.edu.pk/new/dow-lab/>  · <https://chughtailab.com/> |
| Cost effectiveness Does the cost-effectiveness of the intervention favor the intervention or the comparison? | |
| Judgement | Research evidence |
| ○ Favors the comparison ○ Probably favors the comparison ○ Does not favor either the intervention or the comparison ○ Probably favors the intervention ● Favors the intervention ○ Varies ○ No included studies | **Evidence from Source Guideline:**    No evidence from the source guideline is pertinent to this query in original source guideline (2016 American Thyroid Association Guidelines for Diagnosis and Management of Hyperthyroidism and Other Causes of Thyrotoxicosis).  -----------------------------------------------------------------------------------    **Local Evidence:**    Literature search on PubMed found no local studies for answering this question. Our inclusion criteria were adults >18 years of patients with Graves’ Disease in Pakistan/South Asia from the date 1 Jan 2016 till 15 Sept 2021.  No local evidence is pertinent to this query.  ----------------------------------------------------------------------------------------- |
| Equity What would be the impact on health equity? | |
| Judgement | Research evidence |
| ○ Reduced ● Probably reduced ○ Probably no impact ○ Probably increased ○ Increased ○ Varies ○ Don't know | **Evidence from Source Guideline:**    No evidence from the source guideline is pertinent to this query in original source guideline (2016 American Thyroid Association Guidelines for Diagnosis and Management of Hyperthyroidism and Other Causes of Thyrotoxicosis).  -----------------------------------------------------------------------------------  **Local Evidence:**    Literature search on PubMed found no studies with the local context. Our inclusion criteria were adults >18 years of patients with Graves’ Disease in Pakistan/South Asia from the date 1 Jan 2016 till 15 Sept 2021.    No local evidence is pertinent to this query.  ---------------------------------------------------------------------------- |
| Acceptability Is the intervention acceptable to key stakeholders? | |
| Judgement | Research evidence |
| ○ No ○ Probably no ○ Probably yes ● Yes ○ Varies ○ Don't know | **Evidence from Source Guideline:**    No evidence from the source guideline is pertinent to this query in original source guideline (2016 American Thyroid Association Guidelines for Diagnosis and Management of Hyperthyroidism and Other Causes of Thyrotoxicosis).  ------------------------------------------------------------------------  **Local Evidence:**  Literature search on PubMed found no studies with the local context. Our inclusion criteria were adults >18 years of patients with Graves’ Disease in Pakistan/South Asia from the date 1 Jan 2016 till 15 Sept 2021.    No patient advocate input is available.    No direct evidence is pertinent to this query.  ----------------------------------------------------------------------------------------- |
| Feasibility Is the intervention feasible to implement? | |
| Judgement | Research evidence |
| ○ No ○ Probably no ○ Probably yes ● Yes ○ Varies ○ Don't know | **Indirect Evidence:**    No evidence from the source guideline is pertinent to this query in original source guideline (2016 American Thyroid Association Guidelines for Diagnosis and Management of Hyperthyroidism and Other Causes of Thyrotoxicosis).  ---------------------------------------------------------------------------  **Local Evidence:**    Literature search on PubMed found no studies with the local context. Our inclusion criteria were adults >18 years of patients with Graves’ Disease in Pakistan/South Asia from the date 1 Jan 2016 till 15 Sept 2021.  No local evidence is pertinent to this query.  ----------------------------------------------------------------------------------------- |

# Summary of judgementS

|  | **Judgement** | | | | | | |
| --- | --- | --- | --- | --- | --- | --- | --- |
| **Problem** | No | Probably no | Probably yes | **Yes** |  | Varies | Don't know |
| **Desirable Effects** | Trivial | Small | **Moderate** | Large |  | Varies | Don't know |
| **Undesirable Effects** | Large | Moderate | **Small** | Trivial |  | Varies | Don't know |
| **Certainty of evidence** | Very low | **Low** | Moderate | High |  |  | No included studies |
| **Values** | Important uncertainty or variability | Possibly important uncertainty or variability | **Probably no important uncertainty or variability** | No important uncertainty or variability |  |  |  |
| **Balance of effects** | Favors the comparison | Probably favors the comparison | Does not favor either the intervention or the comparison | **Probably favors the intervention** | Favors the intervention | Varies | Don't know |
| **Resources required** | Large costs | **Moderate costs** | Negligible costs and savings | Moderate savings | Large savings | Varies | Don't know |
| **Certainty of evidence of required resources** | Very low | Low | **Moderate** | High |  |  | No included studies |
| **Cost effectiveness** | Favors the comparison | Probably favors the comparison | Does not favor either the intervention or the comparison | Probably favors the intervention | **Favors the intervention** | Varies | No included studies |
| **Equity** | Reduced | **Probably reduced** | Probably no impact | Probably increased | Increased | Varies | Don't know |
| **Acceptability** | No | Probably no | Probably yes | **Yes** |  | Varies | Don't know |
| **Feasibility** | No | Probably no | Probably yes | **Yes** |  | Varies | Don't know |

# Type of recommendation

| Strong recommendation against the intervention | Conditional recommendation against the intervention | Conditional recommendation for either the intervention or the comparison | **Conditional recommendation for the intervention** | Strong recommendation for the intervention |
| --- | --- | --- | --- | --- |
| ○ | ○ | ○ | **●** | ○ |
