# Supplementary material for: GRADE-ADOLOPMENT of hyperthyroidism treatment guidelines for a Pakistani context
Source: BMC Endocr Disord. 2024 Mar 21;24:41. doi: 10.1186/s12902-023-01493-1 (PMC10956339; doi:10.1186/s12902-023-01493-1)
Supplement: Supplementary file 3 — Additional file 3. [file 12902_2023_1493_MOESM3_ESM.docx]

| Supplementary Table 6: Evidence to decision table for recommendation # 18 | |
| --- | --- |
| Question | |
| **Should Liver function tests vs. no labs be used for patients taking MMI/PTU who experience pruritic rash, jaundice, light-colored stool or dark urine, joint pain, abdominal pain or bloating, anorexia, nausea, or fatigue.?** | |
| **Population:** | patients taking MMI/PTU who experience pruritic rash, jaundice, light-coloured stool or dark urine, joint pain, abdominal pain or bloating, anorexia, nausea, or fatigue. |
| **Intervention:** | Liver function tests |
| **Comparison:** | no labs |
| **Main outcomes:** |  |
| **Setting:** | Pakistan, South Asia |
| **Perspective:** |  |
| **Background:** |  |
| **Conflict of interests:** |  |

# Assessment

| Problem Is the problem a priority? | |
| --- | --- |
| Judgement | Research evidence |
| ○ No ○ Probably no ● Probably yes ○ Yes ○ Varies ○ Don't know | In Pakistan, the prevalence of hyperthyroidism is 5.1% and is higher in females than males (1).    Ref:  1. Hussain, A., & Avais, J. (2020). Grave’s Disease: When to Jump for Operative Management. *Archives of Surgical Research*, *1*(4), 12-16. Retrieved from [http://archivessr.com/index.php/asr/article/view/4](http://archivessr.com/index.php/asr/article/view/49)9    In Pakistan, the prevalence of overt and sub-clinical hyperthyroidism is reported to be 5.1% and 5.8%, respectively. Similarly, the prevalence of overt and sub-clinical hypothyroidism is observed at 4.1 and 5.4%, respectively. It is also perceived that the prevalence of both hyperthyroidism and hypothyroidism (subclinical or overt) is higher in females than males (2).    Ref:  2. Iqbal, A., et al. (2016). Prevalence and manifestations of thyroidal dysfunction in central Punjab Pakistan. Sci.Int.(Lahore),28(4),3959-3963. Retrieved from http://www.sci-int.com/pdf/636428150898615705.%20Amir%20Iqbal--ZOO--PU--28-6-16--REVIEWED%20by%20Zahid.pdf |
| Desirable Effects How substantial are the desirable anticipated effects? | |
| Judgement | Research evidence |
| ○ Trivial ○ Small ● Moderate ○ Large ○ Varies ○ Don't know | Literature search on PubMed found 187 article relevant articles. These articles were screened for title and abstract and then 3 extracted articles were screened for full text but all of the studies were not in the local context. Our inclusion criteria were adults >18 years of patients with Graves’ Disease in Pakistan/South Asia from the date 1 Jan 2016 till 15 Sept 2021.    In one case-control study, assessment of carbimazole, propylthiouracil & l-thyroxine for liver markers was done in thyroid patients from Punjab, Pakistan and it was suggested that liver function tests should also be performed in thyroid patients to check any abnormality in liver function (3).    Ref:  3. Siddiqui, M., Anwer, H., Batool, Z., Hasnain, S., Imtiaz, M., Tasneem, A., Fatima, I., Ahmad, S., & Alam, R. (2015). Assessment of Carbimazole, Propylthiouracil & L-Thyroxine for Liver Markers in Thyroid Patients from Punjab, Pakistan. *Journal of applied pharmacy, 7*, 105-113.    ---------------------------------  Indirect Evidence:    No indirect evidence is available in original source guideline (2016 American Thyroid Association Guidelines for Diagnosis and Management of Hyperthyroidism and other Causes of Thyrotoxicosis). |
| Undesirable Effects How substantial are the undesirable anticipated effects? | |
| Judgement | Research evidence |
| ○ Large ○ Moderate ● Small ○ Trivial ○ Varies ○ Don't know | Literature search on PubMed found 187 article relevant articles. These articles were screened for title and abstract and then 3 extracted articles were screened for full text but all of the studies were not in the local context. Our inclusion criteria were adults >18 years of patients with Graves’ Disease in Pakistan/South Asia from the date 1 Jan 2016 till 15 Sept 2021.    A case report of a 30-year-old male patient with Graves’ disease, who developed cholestatic jaundice after Carbimazole therapy for four months. He made a full recovery after the drug was discontinued. The study further elaborated that hepatic toxicity is a rare, but serious side effect of antithyroid medications. Doctors dealing with thyroid patients should be aware of such complications. Routine liver function tests during therapy are not cost-effective but must be performed when this complication is suspected (4).    Ref:  4. Kota, S. K., Meher, L. K., Kota, S. K., Jammula, S., & Modi, K. D. (2013). Carbimazole-induced cholestatic hepatitis in Graves' disease. *Indian journal of endocrinology and metabolism*, *17*(2), 326–328. https://doi.org/10.4103/2230-8210.109660    --------------------------------------------------------------------------  Indirect Evidence:    "Hyperthyroidism can itself cause mildly abnormal liver function tests in up to 30% of patients (5). PTU may cause transient elevations of serum transaminases in up to one-third of patients. Signiﬁcant elevations to 3-fold above the upper limit of normal are seen in up to 4% of patients taking PTU (6), a prevalence higher than with MMI. As previously noted, PTU can also cause fatal hepatic necrosis, leading to the suggestion by some that patients taking this ATD have routine monitoring of their liver function, especially during the ﬁrst 6 months of therapy. A 2009 review of the literature (7) found that PTU hepatotoxicity occurred after a median of 120 days after initiation of therapy.  Routine monitoring of liver function in all patients taking ATDs has not been found to prevent severe hepatotoxicity. If monitoring is employed, the maximum beneﬁt would be for the ﬁrst 120 days of therapy, when the vast majority of instances of hepatotoxicity occur. "    "2016 American Thyroid Association Guidelines for Diagnosis and Management of Hyperthyroidism and Other Causes of Thyrotoxicosis"    Ref:  5. Cooper DS, Rivkees SA 2009 Putting propylthiouracil in perspective. J Clin Endocrinol Metab 94:1881–1882  6. Huang MJ, Liaw YF 1995 Clinical associations between thyroid and liver diseases. J Gastroenterol Hepatol 10: 344–350.  7. Liaw YF, Huang MJ, Fan KD, Li KL, Wu SS, Chen TJ 1993 Hepatic injury during propylthiouracil therapy in patients with hyperthyroidism. A cohort study. Ann Intern Med 118:424–428. |
| Certainty of evidence What is the overall certainty of the evidence of effects? | |
| Judgement | Research evidence |
| ○ Very low ● Low ○ Moderate ○ High ○ No included studies | Literature search on PubMed found 187 article relevant articles. These articles were screened for title and abstract and then 3 extracted articles were screened for full text but all of the studies were not in the local context. Our inclusion criteria were adults >18 years of patients with Graves’ Disease in Pakistan/South Asia from the date 1 Jan 2016 till 15 Sept 2021.    There are no direct comparative studies that compare different intervals and assess them on patient-important outcomes.  ------------------------------------------  Indirect Evidence:    No indirect evidence is available in original source guideline (2016 American Thyroid Association Guidelines for Diagnosis and Management of Hyperthyroidism and other Causes of Thyrotoxicosis). |
| Values Is there important uncertainty about or variability in how much people value the main outcomes? | |
| Judgement | Research evidence |
| ○ Important uncertainty or variability ● Possibly important uncertainty or variability ○ Probably no important uncertainty or variability ○ No important uncertainty or variability | Literature search on PubMed found 187 article relevant articles. These articles were screened for title and abstract and then 3 extracted articles were screened for full text but all of the studies were not in the local context. Our inclusion criteria were adults >18 years of patients with Graves’ Disease in Pakistan/South Asia from the date 1 Jan 2016 till 15 Sept 2021.    No patient advocate input is available.    No direct evidence is available on patient values.  ------------------------------------  Indirect Evidence:    No indirect evidence is available in original source guideline (2016 American Thyroid Association Guidelines for Diagnosis and Management of Hyperthyroidism and other Causes of Thyrotoxicosis). |
| Balance of effects Does the balance between desirable and undesirable effects favor the intervention or the comparison? | |
| Judgement | Research evidence |
| ○ Favors the comparison ○ Probably favors the comparison ○ Does not favor either the intervention or the comparison ● Probably favors the intervention ○ Favors the intervention ○ Varies ○ Don't know | In one case-control study, assessment of carbimazole, propylthiouracil & l-thyroxine for liver markers was done in thyroid patients from Punjab, Pakistan and it was suggested that liver function tests should also be performed in thyroid patients to check any abnormality in liver function because of thyroid disorder (3).    Ref:  3. Siddiqui, M., Anwer, H., Batool, Z., Hasnain, S., Imtiaz, M., Tasneem, A., Fatima, I., Ahmad, S., & Alam, R. (2015). Assessment of Carbimazole, Propylthiouracil & L-Thyroxine for Liver Markers in Thyroid Patients from Punjab, Pakistan. *Journal of applied pharmacy, 7*, 105-113. |
| Resources required How large are the resource requirements (costs)? | |
| Judgement | Research evidence |
| ○ Large costs ● Moderate costs ○ Negligible costs and savings ○ Moderate savings ○ Large savings ○ Varies ○ Don't know | As per telephonic research the cost of LFTs in Pakistan/ South Asia:    Aga Khan University Hospital: 2100/- PKR    Dr. Essa Laboratory and Diagnostic Centre: 1290/- PKR    Chughtai lab: 1650/- PKR    Dow Lab: 700/- PKR  Only SGPT/ALT at AKUH: 600 PKR |
| Certainty of evidence of required resources What is the certainty of the evidence of resource requirements (costs)? | |
| Judgement | Research evidence |
| ○ Very low ○ Low ○ Moderate ● High ○ No included studies | As per google search, the diagnostic Centers testing LFTs:    Aga Khan University Hospital:  https://hospitals.aku.edu/pakistan/medical-and-diagnostics/clinical-labs/Pages/default.aspx    Dr Essa Laboratory and Diagnostic Centre:  https://www.dressalab.com/    Chughtai lab:  https://chughtailab.com/    Dow Lab:  https://www.duhs.edu.pk/new/dow-lab/ |
| Cost effectiveness Does the cost-effectiveness of the intervention favor the intervention or the comparison? | |
| Judgement | Research evidence |
| ○ Favors the comparison ○ Probably favors the comparison ○ Does not favor either the intervention or the comparison ○ Probably favors the intervention ● Favors the intervention ○ Varies ○ No included studies | Literature search on PubMed found 187 article relevant articles. These articles were screened for title and abstract and then 3 extracted articles were screened for full text but all of the studies were not in the local context. Our inclusion criteria were adults >18 years of patients with Graves’ Disease in Pakistan/South Asia from the date 1 Jan 2016 till 15 Sept 2021.    No direct evidence is pertinent to this query.  ------------------------------------  Indirect Evidence:    No indirect evidence is available in original source guideline (2016 American Thyroid Association Guidelines for Diagnosis and Management of Hyperthyroidism and other Causes of Thyrotoxicosis). |
| Equity What would be the impact on health equity? | |
| Judgement | Research evidence |
| ○ Reduced ○ Probably reduced ○ Probably no impact ● Probably increased ○ Increased ○ Varies ○ Don't know | Literature search on PubMed found 187 article relevant articles. These articles were screened for title and abstract and then 3 extracted articles were screened for full text but all of the studies were not in the local context. Our inclusion criteria were adults >18 years of patients with Graves’ Disease in Pakistan/South Asia from the date 1 Jan 2016 till 15 Sept 2021.    No direct evidence is pertinent to this query.  ------------------------------------  Indirect Evidence:    No indirect evidence is available in original source guideline (2016 American Thyroid Association Guidelines for Diagnosis and Management of Hyperthyroidism and other Causes of Thyrotoxicosis). |
| Acceptability Is the intervention acceptable to key stakeholders? | |
| Judgement | Research evidence |
| ○ No ○ Probably no ● Probably yes ○ Yes ○ Varies ○ Don't know | Literature search on PubMed found 187 article relevant articles. These articles were screened for title and abstract and then 3 extracted articles were screened for full text but all of the studies were not in the local context. Our inclusion criteria were adults >18 years of patients with Graves’ Disease in Pakistan/South Asia from the date 1 Jan 2016 till 15 Sept 2021.    No patient advocate input is available.  No direct evidence is pertinent to this query.  ------------------------------------  Indirect Evidence:    No indirect evidence is available in original source guideline (2016 American Thyroid Association Guidelines for Diagnosis and Management of Hyperthyroidism and other Causes of Thyrotoxicosis). |
| Feasibility Is the intervention feasible to implement? | |
| Judgement | Research evidence |
| ○ No ○ Probably no ○ Probably yes ● Yes ○ Varies ○ Don't know | Literature search on PubMed found 187 article relevant articles. These articles were screened for title and abstract and then 3 extracted articles were screened for full text but all of the studies were not in the local context. Our inclusion criteria were adults >18 years of patients with Graves’ Disease in Pakistan/South Asia from the date 1 Jan 2016 till 15 Sept 2021.  No direct evidence is pertinent to this query.  ------------------------------------  Indirect Evidence:    No indirect evidence is available in original source guideline (2016 American Thyroid Association Guidelines for Diagnosis and Management of Hyperthyroidism and other Causes of Thyrotoxicosis). |

# Summary of judgements

|  | **Judgement** | | | | | | |
| --- | --- | --- | --- | --- | --- | --- | --- |
| **Problem** | No | Probably no | **Probably yes** | Yes |  | Varies | Don't know |
| **Desirable Effects** | Trivial | Small | **Moderate** | Large |  | Varies | Don't know |
| **Undesirable Effects** | Large | Moderate | **Small** | Trivial |  | Varies | Don't know |
| **Certainty of evidence** | Very low | **Low** | Moderate | High |  |  | No included studies |
| **Values** | Important uncertainty or variability | **Possibly important uncertainty or variability** | Probably no important uncertainty or variability | No important uncertainty or variability |  |  |  |
| **Balance of effects** | Favors the comparison | Probably favors the comparison | Does not favor either the intervention or the comparison | **Probably favors the intervention** | Favors the intervention | Varies | Don't know |
| **Resources required** | Large costs | **Moderate costs** | Negligible costs and savings | Moderate savings | Large savings | Varies | Don't know |
| **Certainty of evidence of required resources** | Very low | Low | Moderate | **High** |  |  | No included studies |
| **Cost effectiveness** | Favors the comparison | Probably favors the comparison | Does not favor either the intervention or the comparison | Probably favors the intervention | **Favors the intervention** | Varies | No included studies |
| **Equity** | Reduced | Probably reduced | Probably no impact | **Probably increased** | Increased | Varies | Don't know |
| **Acceptability** | No | Probably no | **Probably yes** | Yes |  | Varies | Don't know |
| **Feasibility** | No | Probably no | Probably yes | **Yes** |  | Varies | Don't know |

# Type of recommendation

| Strong recommendation against the intervention | Conditional recommendation against the intervention | Conditional recommendation for either the intervention or the comparison | Conditional recommendation for the intervention | **Strong recommendation for the intervention** |
| --- | --- | --- | --- | --- |
